# Supplementary material for: Identification of diagnostic biomarkers of and immune cell infiltration analysis in bovine respiratory disease
Source: Front Vet Sci. 2025 Mar 5;12:1556676. doi: 10.3389/fvets.2025.1556676 (PMC11921050; doi:10.3389/fvets.2025.1556676)
Supplement: SUPPLEMENTARY TABLE 8 — GSEA analysis results of key genes. [file Table_8.docx]

**Additional table 10： Nucleotide sequences of the primers used for Real-time fluorescence quantitative PCR**

| Genes | Direction | Primer sequence (5’-3’) | Amplification size |
| --- | --- | --- | --- |
| ADGRG3 | F | CGCTGTGACCACCTGACCTTCT | 91bp |
|  | R | AGGAGCCTCGGCATTTCTGGAA |  |
| CDKN1A | F | ACCACTTGGACCTGTCGCTGTC | 115bp |
|  | R | ATGCTGGTCTGCCGCCGTTT |  |
| CA4 | F | CGTCAACATCGTCACAGCCAAG | 113bp |
|  | R | ACCATCACTGTATGCCCGTTGT |  |
| GGT5 | F | CTGTGGCTGGGCTTTGACCT | 121bp |
|  | R | CCTTCTGAACCTCCTGGCTGAA |  |
| SLC26A8 | F | TTGACGCTGGCATCACTAAGGC | 108bp |
|  | R | ACTCGGAGGACTCTGGCAACTT |  |
| GAPDH | F | ACAGTCAAGGCAGAGAACGG | 98bp |
|  | R | CCAGCATCACCCCACTTGAT |  |

**Real-time fluorescence quantitative reaction program**

| Procedure | Temperature | Time | Cycles |
| --- | --- | --- | --- |
| Preincubation | 95℃ | 600 s | 1 |
|  | 95℃ | 10 s |  |
| 3 Step Amplification | 63℃ | 20 s | 35 |
|  | 72℃ | 15 s |  |
|  | 95℃ | 10 s |  |
| Melting | 65℃ | 60 s | 1 |
|  | 97℃ | 1 s |  |
| Cooling | 97℃ | 30 s | 1 |
