# Supplementary material for: Identification of diagnostic biomarkers of and immune cell infiltration analysis in bovine respiratory disease
Source: Front Vet Sci. 2025 Mar 5;12:1556676. doi: 10.3389/fvets.2025.1556676 (PMC11921050; doi:10.3389/fvets.2025.1556676)
Supplement: Supplementary file 14 [file Table_11.docx]

Identification of diagnostic biomarkers of and immune cell infiltration analysis in bovine respiratory disease

Hui Sheng^1†^, Junxing Zhang^2†^, Xiaodi Shi^1^, Long Zhang^1,2^, Dawei Yao^1^, Peipei Zhang^1^, Yupeng Li^1^, Jinlong Zhang^1^, Xiaofei Guo^1^*, Xiaosheng Zhang^1^*

^1^ Tianjin Key Laboratory of Animal Molecular Breeding and Biotechnology, Tianjin Engineering Research Center of Animal Healthy Farming, Institute of Animal Science and Veterinary, Tianjin Academy of Agricultural Sciences, Tianjin 300381, People's Republic of China.

^2^ College of Animal Science and Veterinary Medicine, Tianjin Agricultural University, Tianjin 300384, China.

^†^ Hui Sheng and Junxing Zhang contributed equally to this work.

*Correspondence: Xiaosheng Zhang [(zhangxs0221@126.com)](mailto:(mayun_666@126.com);); Xiaofei Guo [(Guoxfnongda@163.com)](mailto:(mayun_666@126.com);)

**Abstract:** Bovine respiratory disease (BRD) is a prevalent and costly condition in the cattle industry, impacting long-term productivity, antibioticusage, and global food safety. Thus, identifying reliable biomarkers for BRD is crucial for early diagnosis, effective treatment, and monitoring therapeutic outcomes. This study identified differentially expressed genes (DEGs) associated with BRD by analyzing a blood RNA-seq expression dataset associated with BRD, and conducted a Kyoto Encyclopedia of Genes and Genomes (KEGG) approach enrichment and Gene Ontology (GO) annotation analysis on these DEGs. Meanwhile, the key modules related to BRD were screened by weighted gene co-expression network analysis (WGCNA), and the genes in the module were intersected with DEGs. Subequently, least absolute shrinkage and selection operator (LASSO) and random forest (RF) analysis were employed to identify potential biomarkers. Finally, gene set enrichment analysis (GSEA) was performed to explore the potential mechanisms of the identified biomarkers, and their diagnostic significance was assessed using receiver operator characteristic (ROC) curve analysis and real-time fluorescent quantitative PCR (RT-qPCR). In addition, immune cell infiltration in BRD was evaluated using the CIBERSORT algorithm and the correlation between biomarkers and immune cell infiltration was analyzed. The results showed that a total of 1,097 DEG were screened. GO and KEGG analysis showed that DEGs was mainly enriched in inflammatory response, defense response, Complement and coagulation cascades and Antigen processing and presentation pathways. WGCNA analysis determined that the cyan module had the highest correlation with BRD. A total of 833 overlapping genes were identified through Veen analysis of the differential and WGCNA results. Lasso and RF analyses identified five potential biomarkers for BRD. RT-qPCR testing and data set analysis showed that the expression levels of these five potential biomarkers in nasal mucus and blood of BRD cattle were significantly higher than those of healthy cattle. In addition, ROC curve analysis showed that potential biomarkers had high diagnostic value. GSEA analysis revealed that potential biomarkers are mainly involved in Neutrophil extracellular trap formation, Complement and coagulation cascades, T cell receptor signaling pathway, B cell receptor signaling pathway, Fc gamma R-mediated phagocytosis and IL-17 signaling pathway. The results from the CIBERSORT algorithm demonstrated a significant difference in immune cell composition between the BRD group and the healthy group, indicating that the diagnostic biomarkers were closely associated with immune cells. In conclusion, this study identified ADGRG3, CDKN1A, CA4, GGT5, and SLC26A8 as potential diagnostic markers for BRD, providing significant insights for the deelopment of new immunotherapy targets and improving disease prevention and treatment strategies.

**Keywords:** Bovine respiratory disease; lifetime productivity; potential biomarkers; immune infiltration; CIBERSORT

1. Introduction

BRD, also known as calf pneumonia, is the main cause of calf morbidity and death, resulting in huge economic losses and damage to animal welfare [1-3]. BRD is caused by a variety of factors and is associated with infection of cattle with bacterial (*Mycoplasma bovis*, *Mannheimia haemolytica*, *Pasteurella multocida*, *Haemophilus*) and viral (*Bovine respiratory syncytial virus* (BRSV), *Bovine herpesvirus type 1* (BHV-1), *Bovine viral diarrhea virus* (BVDV), *Bovine Parainfluenza Virus type 3* (PI-3), and *Bovine Coronavirus* (BCoV) pathogens), which occur when cattle exhibit an inadequate stress resonse [1, 4, 5]. Among them, BRSV is the main cause of BRD in calves (≤ 1 year old). BRSV infection can inhibit the immune defense mechanism of the host, resulting in replication, inhalation and colonization of *M. haemolytica* in the upper respiratory system of cattle, and the host infection of the virus does not show obvious symptoms [6].

Survey data provided by the National Animal Health Monitoring System (NAHMS) indicated that 16.2% of feedlot cattle were affected by BRD [7]. In beef farms and calves from birth to weaning, the prevalence of BRD is about 20% [8]. In addition, BRD is responsible not only for the deaths of 1/4 pre-weaned dairy calf and half of post-weaning dairy calf, but also for about half of the deaths of beef cattle on farms [2, 3, 9]. Taking into account the differences in the environment and management of calves on farms, the researchers speculated that the proportion of cattle with BRD may be much higher than the data assessed by NAHMS. BRD-induced deaths represent a direct loss, whereas the greater economic impact arises from reduced production performance due to repeated treatments and lung lesions [10-12]. Studies have found that beef cattle with BRD result in reduced growth rates as well as lower carcass quality at slaughter [12-14]. Dairy cows suffering from BRD will lead to an increase in the age of first calving, a decrease in first birth and survival rate, a decrease in lactation yield and a decrease in the life span of dairy cows [13, 15, 16]. In the United States, the annual economic loss caused by BRD affecting cattle production performance may exceed 2 billion US dollars [17].

At present, the standard method of BRD on-site detection is a scoring system based on visual clinical diagnosis (VCD), which depends on the observation of rectal temperature, respiratory rate, cough, nasal secretions and so on [18, 19]. However, most clinical symptoms have low sensitivity and specificity for the assessment of BRD, and calves with subclinical BRD cannot be identified [18, 20, 21]. Furthrmore, different examiners have different diagnostic results of the disease, resulting in economic losses and extensive use of antibiotics [22, 23]. It is reported that in the dairy cow population, the diagnostic sensitivity of the VCD scoring system is 77%-100%, and the screening sensitivity is 46 -77%, indicating that about 23% of the infected or suspected infected animals have not been detected. In addition, the average specificity of this method was 46%-92%, indicating that 8%-54% of healthy cattle received unnecessary treatment [24-26]. Therefore, it is very important to reveal the molecular mechanism of BRD and identify the biomarkers of BRD in order to reduce the incidence and the use of antibiotics.

With the rapid development of gene chip and high-throughput sequencing technology, the use of bioinformatics analysis methods to explore potential biomarkers and their complex mechanisms of disease has been widely used. Based on the GSE162156 dataset, this study used a comprehensive strategy of differential expression analysis, co-expression analysis, machine learning analysis and RT-qPCR detection to screen and identify potential biomarkers associated with BRD. In addition, we investigated the correlation between potential biomarkers and infiltrating immune cells using the CIBERSORT algorithm, providing insights to better understand the molecular immune mechanisms underlying BRD and the development of its immune-targeted therapies.

1. Materials and methods

2.1 Sample collection and cytological testing.

Nasal mucus and blood samples were collected from three healthy dairy cows and three sick dairy cows in a large-scale breeding farm. RNA was extracted and reverse transcribed into cDNA for RT-qPCR to detect changes in the expression of target genes.. In addition, changes in neutrophils in nasal mucus cells of healthy and sick cattle were identified based on Giemsa staining. All the experiments were conducted in strict accordance with the recommendations in the guidelines for Animal Protection and Utilization of Tianjin Academy of Agricultural Sciences and approved by the Animal Welfare Committee of Tianjin Academy of Agricultural Sciences.

2.2 Data processing

In this study, the training dataset GSE162156 and the verification dataset GSE150706 are downloaded from GEO database. The GSE162156 dataset includes 18 BRD samples and 18 healthy samples, while the GSE150706 dataset contains 11 BRD samples and 15 health samples [27]. First, FastQC was used to perform quality statistics on the raw data in fastq format, MultiQc was used to integrate the FastQC results, trim_galore was utilized to remove the data with low quality values, and the reads that contained a percentage of N greater than 5% as well as those that contained joints were removed. Second, the cattle reference genome sequence file and annotation file were downloaded from the Ensembl website (https://mart.ensembl.org/index.html), and hisat2 was used to build an index and align the data. Finally, featureCounts was used to count reads and obtain the original expression matrix.

2.3 Identification of DEGs

In this study, BRD samples and healthy samples were analyzed for differential expression using the limma package of R software, and differentially expressed genes were screened using *P* < 0.05 and |log2 fold change (FC)| ≥ 1 as criteria [28, 29]. Volcano mapping of differential genes using the ggplot2 package [30-32]. The Pheatmap package was used to generate a heatmap of the top 200 upregulated and downregulated differentially expressed genes [33, 34].

2.4 Functional enrichment analysis of differential genes

GO and KEGG analyses of differential genes using the clusterProfiler package in R software were used to explore the biological functions of differential genes [35, 36]. Significant enrichment in the GO terms and KEGG pathway was considered when *P* < 0.05.

2.5 Weighted gene co-expression network analysis

WGCNA is a systematic biological method, which is usually used to identify and screen disease markers in organisms. In this study, based on the scale-free topology criterion, the weighted gene co-expression network was analyzed by using WGCNA package in R software [37]. First, check the integrity of the data using the goodSamplesGenes function of the WGCNA package. Second, the pickSoftThreshold function was used to select the optimal soft threshold. The adjacency matrix is constructed by calculating Pearson correlation coefficient, and the adjacency matrix is transformed into topological overlap matrix (TOM). Then, the samples are clustered hierarchically based on the dissimilarity degree of TOM matrix, and all genes are divided into modules. The minimum number of genes in each module was limited to 30, and a merging threshold of 0.25 was used to merge similar modules. Finally, based on the gene significance (GS) value and module member (MM) value to measure the relationship between gene modules and BRD, and finally determine the key modules.

2.6 PPI network construction

We identified common genes DGEs and key module genes by Veen analysis. Subsequently, the Protein-Protein Interaction (PPI) network was constructed for these identified genes using the STRING website (https://cn.string-db.org/), and the results were visualized using Cytoscape software.

2.7 Identification of potential biomarkers.

LASSO is a regression analysis algorithm that combines variable selection and regularization, which can improve the prediction accuracy [38]. RF is a machine learning algorithm based on decision tree theory, which is widely used in sample training and prediction [39]. In this study, the glmnet package of R software was used to perform Lasso analysis on the common genes identified previously [40]. First, intersection validation was performed to set the alpha value to 1 and nfolds to 10. Subsequently, the dataset is divided into a training set and a validation set, the Lasso model is trained on the training set, and then the performance of the model is evaluated on the validation set. RF analysis of common genes was performed using the randomForest package of R software [41]. The dataset was divided into a training set (70% of the data) and a test set (30% of the data). The parameter ntree was set to 180, and the 'importance' function was used to obtain potential biomarkers with high importance. Veen analysis was used to find common genes between LASSO and RF results, defined as potential biomarkers.

2.8 ROC assessment and RT-qPCR assay

In this study, we first evaluated the differential expression of potential biomarkers in BRD samples and healthy samples in GSE162156 data sets. Then, the ROC curve of potential biomarkers was drawn by using the "pROC" package of R software, and the area under the curve (AUC) was calculated to determine the accuracy of potential biomarkers as a diagnostic gene [42, 43]. The closer the AUC value is to 1, the greater the diagnostic value is [44-46]. In addition, the GSE150706 dataset was used to verify the expression level and diagnostic value of potential biomarkers in BRD samples and healthy samples. Finally, total RNA was extracted from bovine blood and nasal mucus samples using TRIZOL reagent (Invitgen, USA). The first-strand cDNA was prepared using PrimeScriptII First-Strand cDNA Synthesis Kit (Takara, Dalian, China). RT-qPCR was performed on a LightCycler® 96 instrument (Roche, Germany) using a All-in-One™ qRT-PCR mixture (Genocopoeia, Guangzhou, China) to detect the expression level of mRNA. In the RT-qPCR experiment of nasal mucus and blood samples, GAPDH mRNA was used as the basic level of endogenous control, and the relative expression level of the gene was calculated using the 2^-ΔΔCt^ method. When *P* < 0.05, it was considered to be statistically significant. All primer information used and RT-qPCR program information are listed in Additional table 10.

2.9 Gene set enrichment analysis

GSEA is often used to analyze and explain changes in pathways and biological processes in expressive data sets [47]. In this study, GSEA analysis of a single potential biomarkers was carried out by using the clusterProfiler package of R software to further determine the potential function of the potential biomarkers associated with BRD.

2.10 CIBERSORT

CIBERSORT is a machine learning algorithm that can analyze the proportion of immune cells in tissue samples [48]. In this study, the 'CiberSort' package of R software was used to analyze the data of the BRD group and the healthy group. The analysis resulted in an expression matrix of 22 immune cell infiltrations, and a bar graph was used to quantify the percentage of each immune cell type in the sample [49]. In addition, based on the matrix of immune cell infiltration obtained by the CIBERSORT algorithm, the Spearman function was used to calculate the correlation coefficient between the gene expression value (x) and the proportion of various immune cell cell types (y) [50].

2.11 Statistical analysis

In this study, nonparametric test or t test was used to analyze the statistical significance of gene expression in BRD samples and healthy samples [51, 52]. Statistical analysis is carried out using the 'ggpubr' package' of R software, and the 'ggplot2' package is used to generate images [30-32, 53].


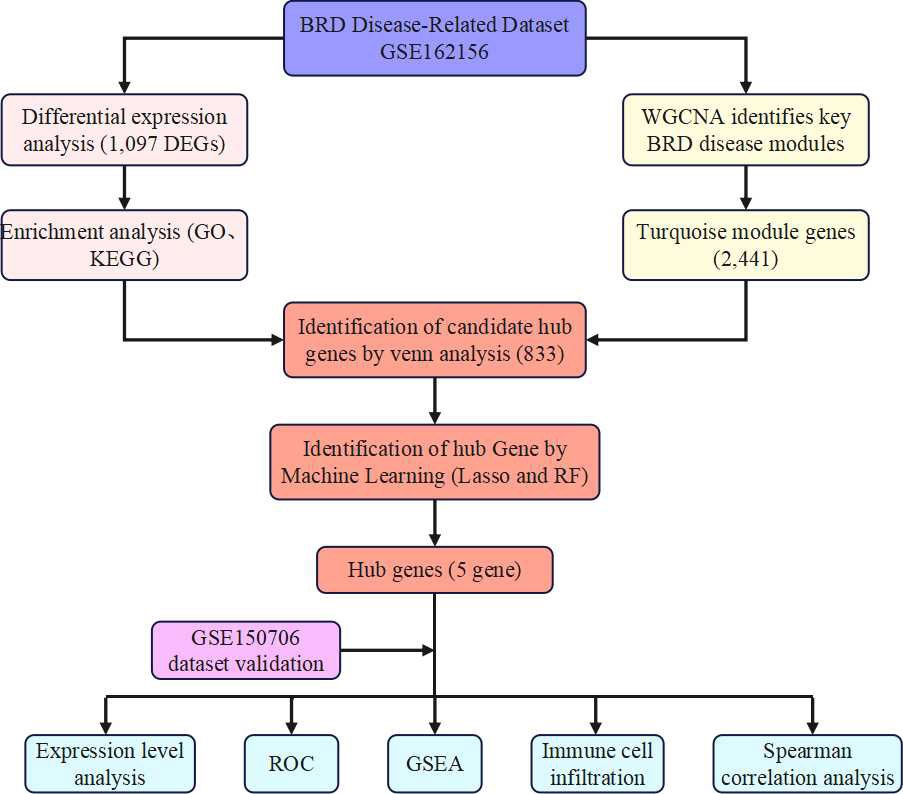


**Figure 1.** The analysis flow chart of this study.

3. Results

3.1 Identification of differentially expressed genes

The flowchart of this study is shown in Figure 1. According to the screening criteria, we identified 1,097 differentially expressed genes in GSE162156 data set, including 653 up-regulated genes and 444 down-regulated genes (Figure 2A, Additional table 1,2). Figure 2B shows the heat map of the first 200 differential genes up-regulated and 200 down-regulated.


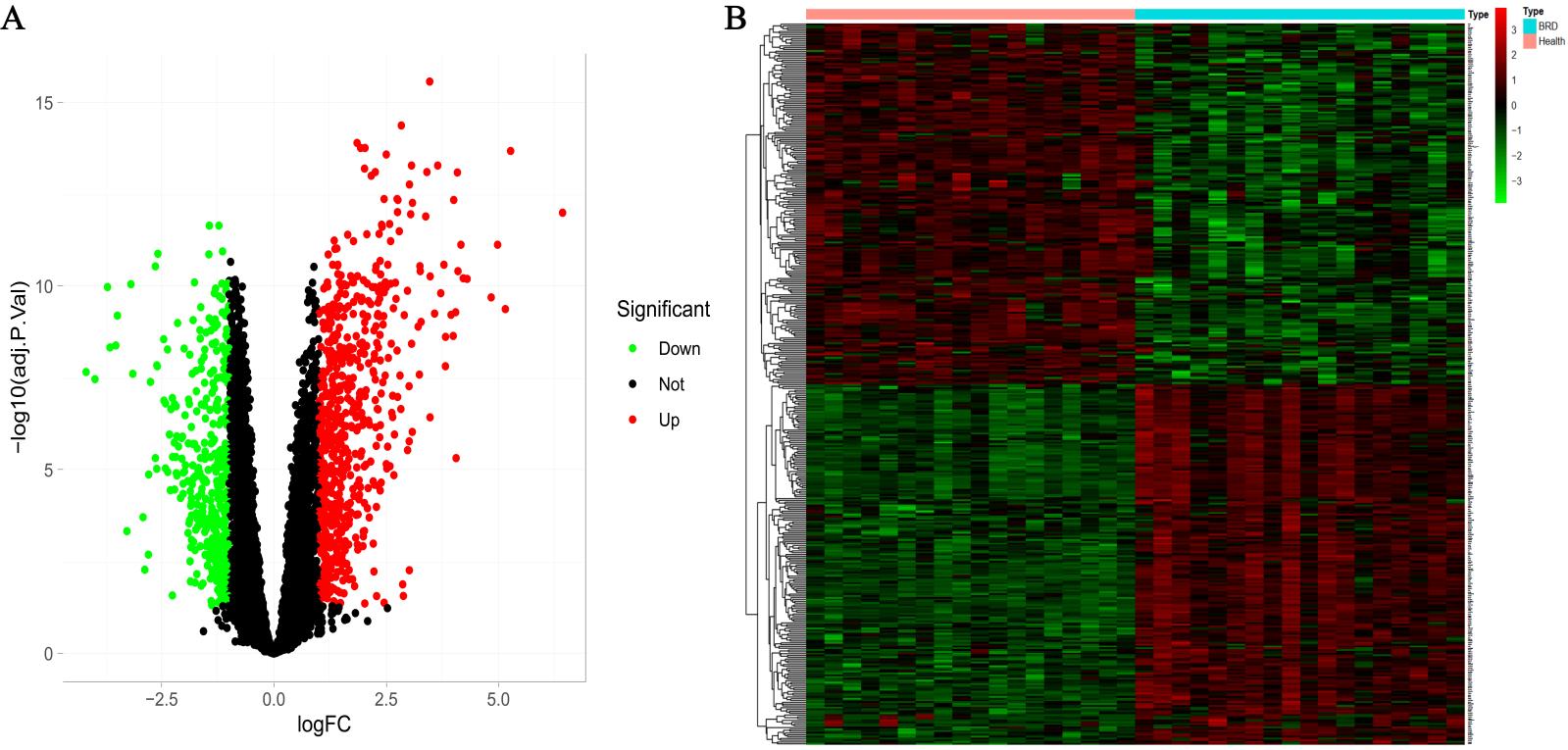


**Figure 2.** Differential expression analysis between BRD and healthy groups. (A) Volcanic map of differentially expressed genes. The screening threshold was set at *P* < 0.05, |log FC|> 1. (B) The heat map of differentially expressed genes.

3.2 Functional enrichment analysis of differentially expressed genes

In this study, GO and KEGG pathways were analyzed to study the biological function of DEGs. The results of Biological Process analysis showed that the differential genes were mainly enriched in inflammatory response, defense response and negative regulation of immune effector process (Figure 3A, Additional table 3). Cell Component analysis showed that the differential genes were enriched in extracellular space, plasma membrane region, membrane raft and membrane microdomain (Figure 3A, Additional table 3). Molecular Function analysis showed that the differential genes were enriched in calcium ion binding, antioxidant activity and G protein-coupled receptor activity (Figure 3A, Additional table 3). KEGG analysis showed that the differential genes were mainly enriched in Complement and coagulation cascades, Cytokine-cytokine receptor interaction, Calcium signaling pathway, Chemokine signaling pathway, Focal adhesion, PI3K-Akt signaling pathway and Antigen processing and presentation (Figure 3B, Additional table 3).


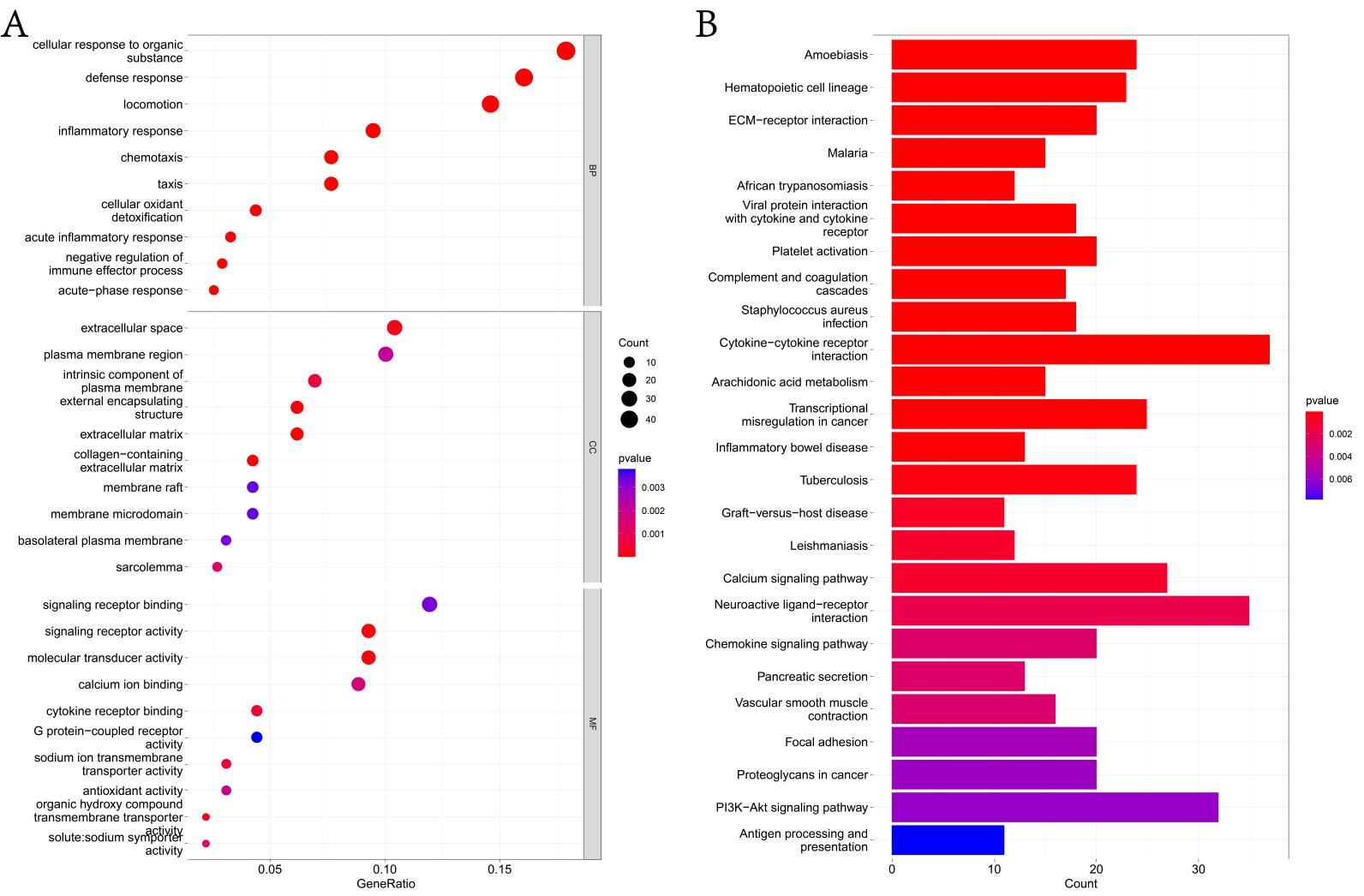


**Figure 3.** Functional enrichment analysis of DEGs. (A) The GO analysis results of DEGs, list the top 10 most important enrichment pathways in BP, CC and MF analyses. (B) KEGG analysis of DEGs list the top 25 most important enrichment pathways in the results.

3.3 Weighted gene co-expression network analysis

In this study, a scale-free co-expression network was constructed using WGCNA to determine the modules most related to BRD. When R^2^ = 0.8, the soft threshold power is determined to be 9 (Figure 4A). The clustering tree of BRD group and healthy group was generated by WGCNA analysis (Figure 4B), and 13 gene co-expression modules were obtained (Figure 4C, Additional table 4). Among them, the turquoise module, which contains 2,441 genes, showed the most significant correlation with BRD (R=0.93, P=3e^-16^). Veen analysis showed that 833 genes in the turquoise module overlap with the DEGs gene, and these overlapping genes will be further used for analysis and identification (Figure 4D). Figure 4E shows that a PPI interaction network of overlapping genes was constructed using the joint analysis of the STRING website and Cytoscape software, clarifying the regulatory relationship between these genes.


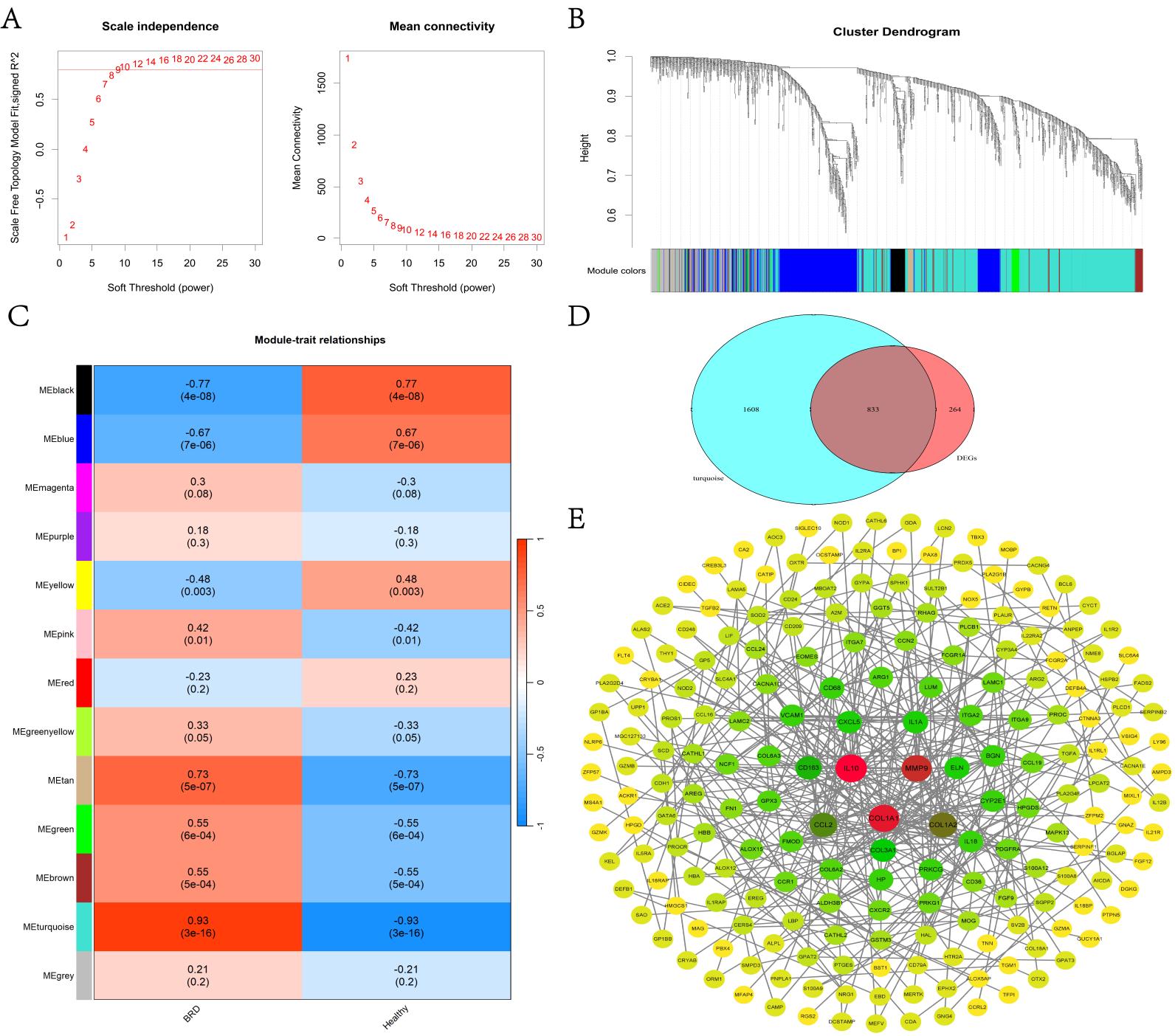


**Figure 4.** WGCNA analysis and identification of candidate potential biomarkers in BRD group and healthy group. (A) Determine the WGCNA soft threshold power (left) and average connectivity (right). (B) Clustering tree diagram for WGCNA. (C) Heat map of the relationship between modules and traits. (D) The gene in turquoise module and the Veen analysis results of DEGs. (E) Construction of overlapping gene PPI network.

3.4 Identification of potential biomarkers based on Machine Learning algorithm

In this study, two machine learning algorithms were utilized to screen key biodiagnostic marker genes for BRD from 833 candidate potential biomarkers. The results showed that 20 possible marker genes were identified by Lasso regression algorithm and 37 by random forest method (Figure 5A-5D, Additional table 5,6). We performed Veen analysis on the results of both algorithms and finally identified five potential biomarkers, including ADGRG3, CDKN1A, CA4, GGT5 and SLC26A8 (Figure 5E).


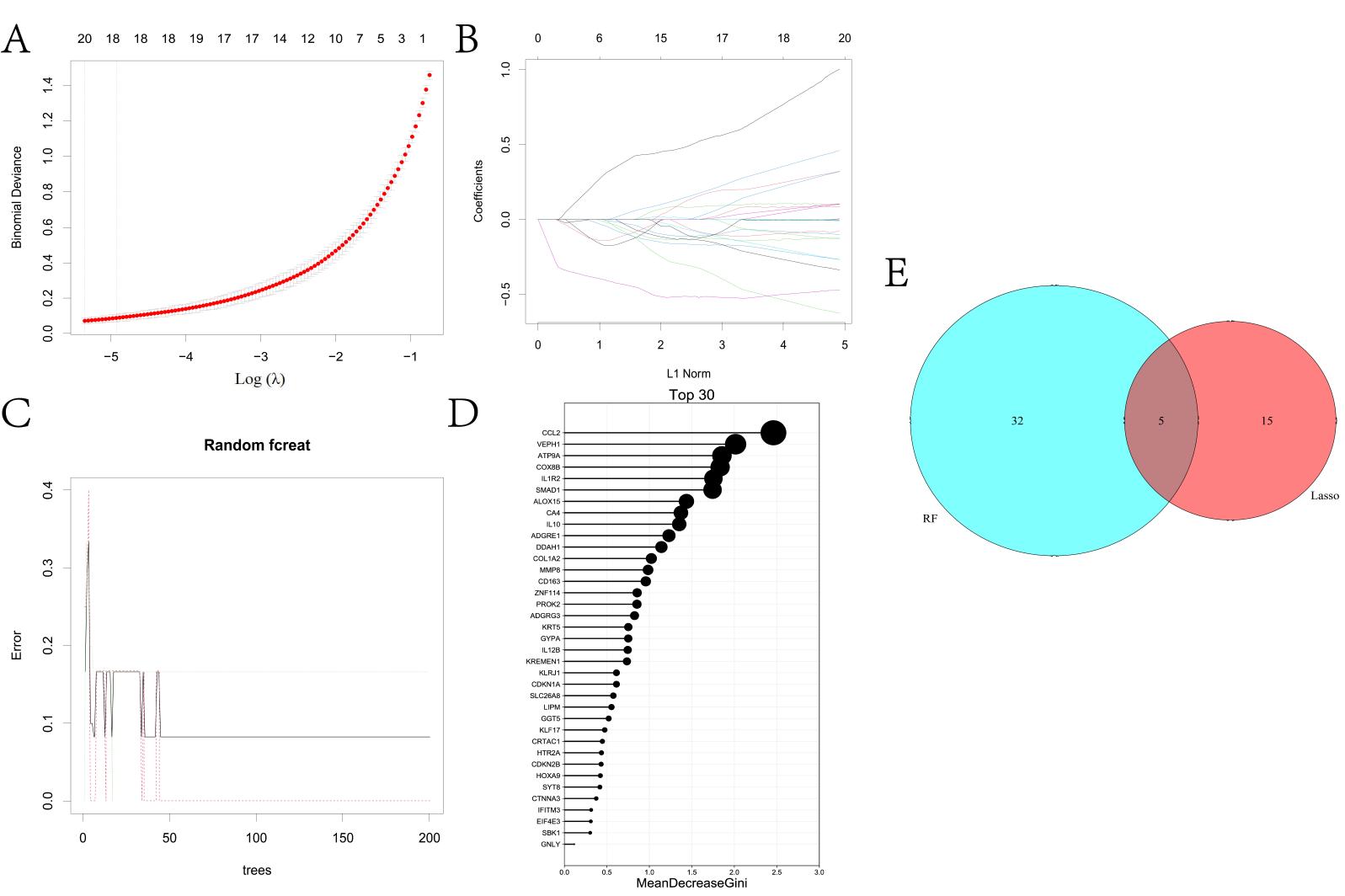


**Figure 5.** To identify the key potential biomarkers of BRD. (A,B) Results of Lasso regression analysis. (C,D) Results of random forest analysis. (E) The overlapping genes in the results of Lasso regression analysis and random forest analysis were determined.

3.5 Analysis of expression level of potential biomarkers in BRD and evaluation of its diagnostic value

In this study, box plot was used to determine the expression level of 5 potential biomarkers in BRD group and healthy group. The results showed that the expression levels of ADGRG3, CDKN1A, CA4, GGT5 and SLC26A8 genes in BRD group were significantly higher than those in healthy group (Figure 6A), and this result was also verified in external data sets GSE150706 (Figure 6B, Additional table 7). Then, the accuracy and specificity of 5 potential biomarkers as diagnostic genes for BRD were determined by ROC curve analysis. The results showed that in the training dataset GSE162156 and the validation dataset GSE150706, the AUC values of the five potential biomarkers were all greater than 0.95, and the True Positive Rate (TPR) of the ordinate (representing sensitivity) and the False Positive Rate (FPR) of the abscissa (representing specificity) were both close to 1 (Additional Figure 3). Theory shows that the closer the AUC value is to 1, the larger the area under the curve, indicating that the higher the accuracy of the prediction model. In addition, RT-qPCR testing of nasal mucus and blood samples from healthy and sick cattle showed that the expression level of potential biomarkers was significantly increased in nasal mucus and blood of cattle with BRD (Figure 6C, 6D). The results show that the screened potential biomarkers are of high value in the diagnosis of BRD.


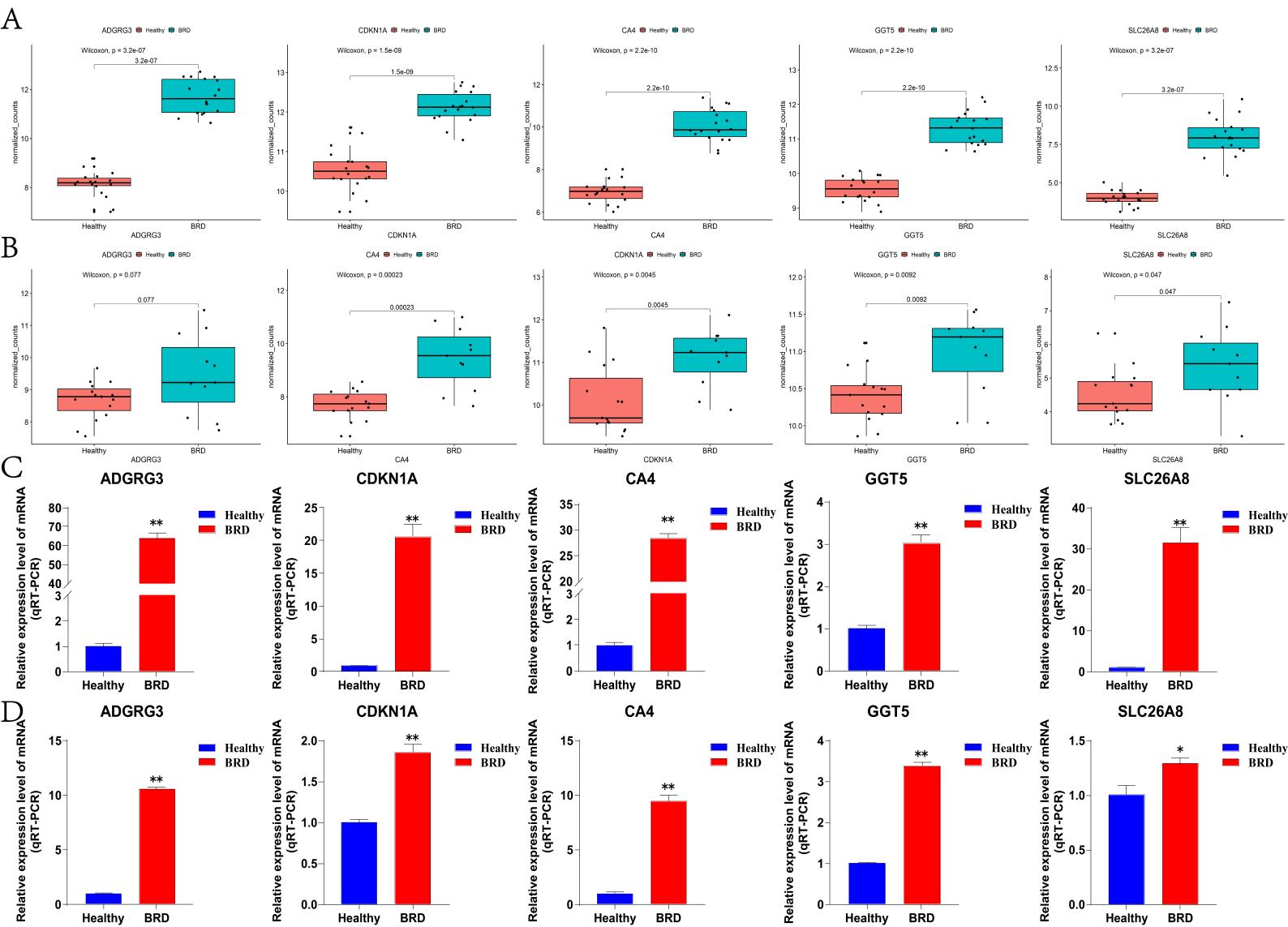


**Figure 6.** The expression level of potential biomarkers was detected. (A) The GSE162156 dataset analyzed potential biomarkers expression levels. (B) The GSE150706 dataset analyzed potential biomarkers expression levels. (C) RT-qPCR was used to detect the expression level of potential biomarkers in bovine nasal mucus samples. (D) RT-qPCR was used to detect the expression level of potential biomarkers in bovine blood samples.

3.6 GSEA analysis of the potential biomarkers.

We studied the specific role of potential biomarkers by GSEA. The results showed that in the training dataset GSE162156, genes showing positive correlation with potential biomarkers were mainly enriched in NOD-like receptor signaling pathway, Neutrophil extracellular trap formation, Necroptosis, Complement and coagulation cascades, T cell receptor signaling pathway, B cell receptor signaling pathway, Lysosome, Fc gamma R-mediated phagocytosis, Th1 and Th2 cell differentiation and IL-17 signaling pathway (Figure 7, Additional table 8).


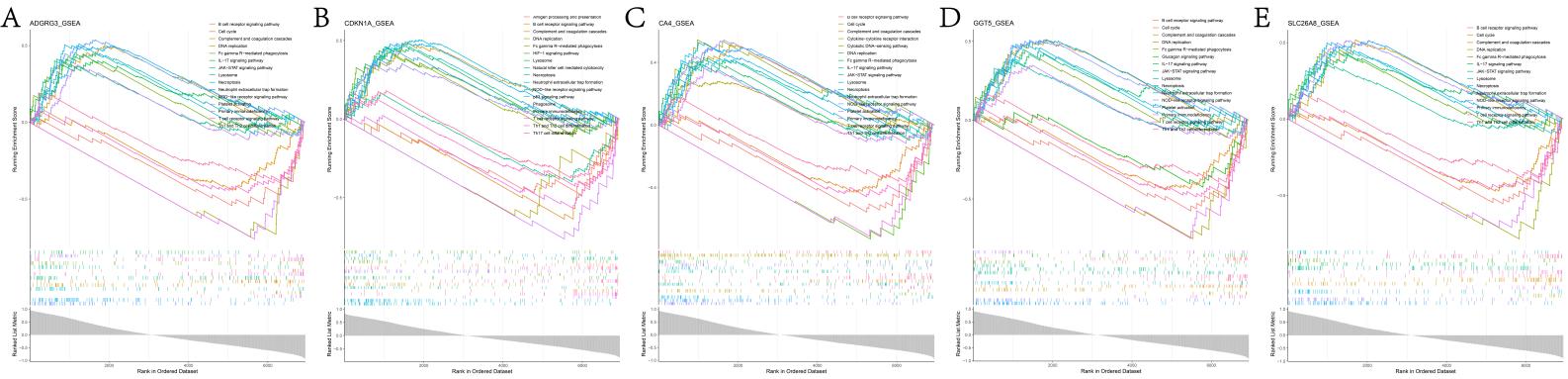


**Figure 7.** Results of GSEA enrichment analysis of genes positively associated with (A) ADGRG3, (B) CDKN1A, (C) CA4, (D) GGT5 and (E) SLC26A8L in the GSE162156 dataset.

3.7 Immune cell infiltration analysis

In this study, CIBERSORT algorithm was used to analyze the changes of the proportion of immune cells in BRD samples and healthy samples (Additional table 9). The results showed that the expressions of B cells memory, T cells CD8 and T cells CD4 naive were higher in healthy samples, while B cells naive, Monocytes and Neutrophils were higher in BRD samples (Figure. 8A). Cytological microscopy also showed that the cells in the nasal mucus of healthy cattle were normal nasal mucosa epithelial cells (NECs) with complete shape, clear and independent nuclei. In the inflammatory group, the proportion of polymorphonuclear leukocytes (PMNs) was higher, and there were fewer normal NECs. The relative heat map of immune cells showed that T cells CD8 was positively correlated with T cells regulatory (Tregs) and B cells memory, and negatively correlated with B cells naive, Neutrophils, T cells CD4 memory activated and Monocytes. B cells memory was positively correlated with T cells CD4 naive and negatively correlated with B cells naive, Neutrophils and Monocytes. T cells CD4 naive was negatively correlated with B cells naive and Monocytes, while T cells regulatory (Tregs) was negatively correlated with Neutrophils and T cells CD4 memory activated. Monocytes was positively correlated with B cells naive, Macrophages M0 and T cells CD4 memory activated, T cells gamma delta was positively correlated with T cells follicular helper and T cells CD4 memory activated, and B cells naive was positively correlated with Neutrophils (Figure. 8B). The difference analysis of immunocyte infiltration between BRD samples and healthy samples is shown in Figure 8C. Compared with the healthy group, the levels of Neutrophils, Monocytes, T cells CD4 memory activated, Plasma cells and B cells naive in BRD group were higher, while the levels of B cells memory, T cells CD8, T cells CD4 naive, T cells regulatory (Tregs) and NK cells resting were lower. In short, there was a significant difference in immune cell infiltration between BRD group and control group.


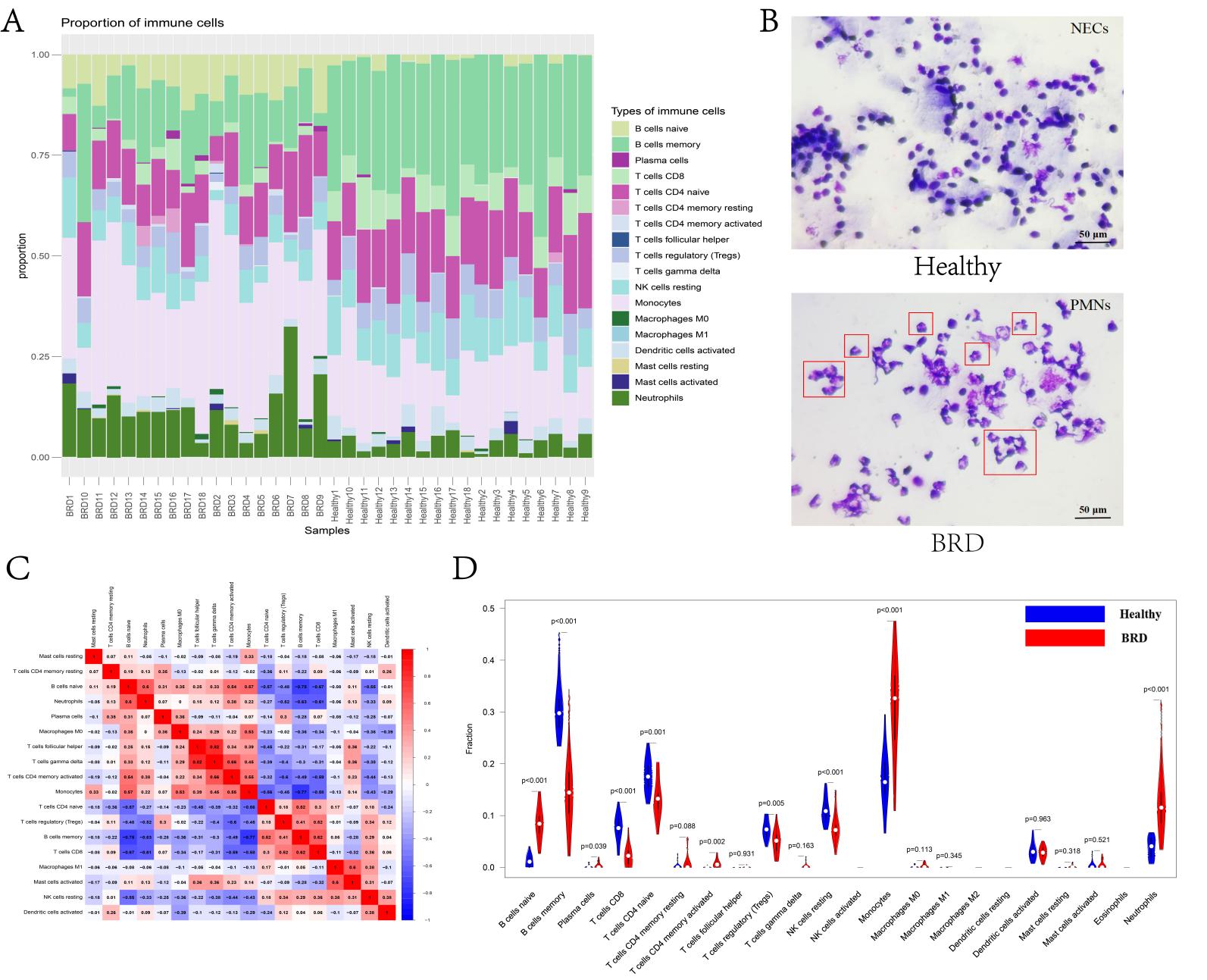


**Figure 8.** Immune cell infiltration analysis. (A) The bar chart shows the proportion of immune cells in different samples. (B) Identification of bovine nasal mucus samples by giemsa staining. (C) The correlation heat map between immune cells. (D) The violin chart shows the difference in immune infiltration between BRD samples and healthy samples.

3.8 Relationship of biomarkers with infiltrating immune cells

In this study, we analyzed the relationship between potential biomarkers and infiltrating immune cells. The results showed that the expression of ADGRG3 (Figure 9A), CDKN1A (Figure 9B), CA4 (Figure 9C), GGT5 (Figure 9D) and SLC26A8 (Figure 9E) genes was positively correlated with the levels of B cells naive, Neutrophils, Monocytes and T cells CD4 memory activated, and negatively correlated with the expression of NK cells resting, T cells regulatory (Tregs), T cells CD4 naive, T cells CD8 and B cells memory (Figure 9). In addition, the expression of CDKN1A gene was positively correlated with the level of T cells CD4 memory resting (Figure 9B), the expression of GGT5 gene was positively correlated with the level of Macrophages M0 and T cells gamma delta (Figure 9D), and the expression of SLC26A8 gene was positively correlated with the level of Macrophages M0, T cells gamma delta and T cells follicular helper (Figure 9E).


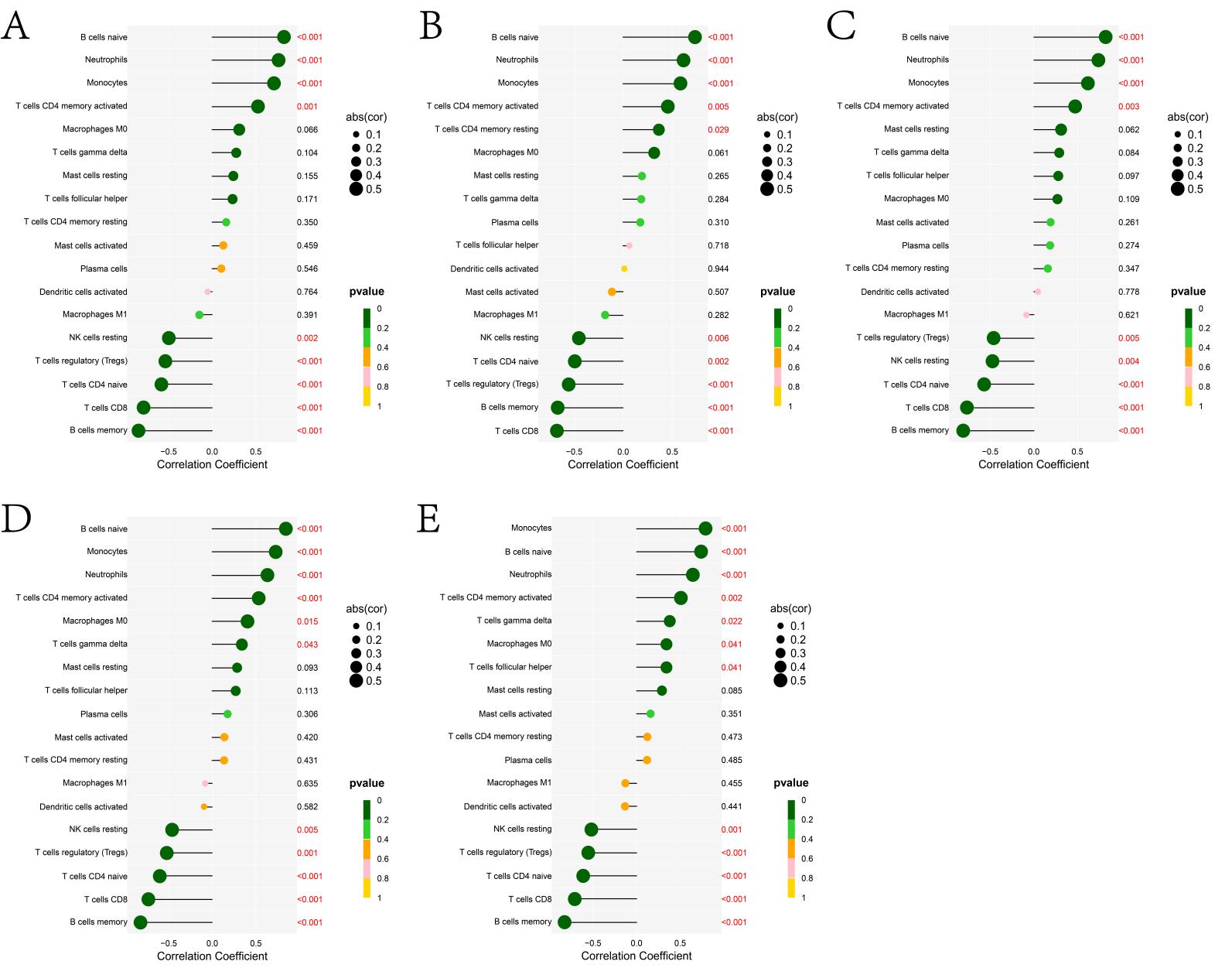


**Figure 9.** Lollipop plots of the correlation between (A) ADGRG3, (B) CDKN1A, (C) CA4, (D) GGT5, and (E) SLC26A8 and immune cells. In the picture, there is a negative correlation on the left and a positive correlation on the right. The larger the dot, the higher the correlation.

4. Discussion

BRD is a respiratory disease caused by bacteria and viruses, which can cause huge economic losses to beef cattle and dairy cattle. therefore, accurate diagnosis is essential for initiating appropriate treatment. RNA-Seq-based transcriptomics can reeal the comprehensive mechanisms of the host response to infection and identify disease-associated genetic markers and their enrichment pathways throughdifferential gene expression analysis [54-57]. Studies have shown differential expression of genes associated with innate immunity in peripheral blood leukocytes of cattle experimentally or naturally infected with BRD [51, 58, 59]. Similarly, differentially expressed genes were continuously enriched in pathways related to innate immunity in bovine bronchial lymphoid tissue infected with a single BRD pathogen [60, 61]. In addition, before cattle were finally diagnosed with BRD and showed clinical symptoms, researchers had detected the differential expression of genes involved in the regulation of inflammation, and the changes in the expression of these genes were related to the severity of the disease [56, 57, 62]. The results show that the identification of disease biomarkers by difference analysis is of great significance for early disease diagnosis and finding new treatment methods.

Blood transcriptomics can detect the level of gene expression in various systems of the body, provide new insights for the identification of system molecular biomarkers, and become a method for early diagnosis of complex infectious diseases [62, 63]. In this study, DEGs in the blood of cattle with BRD and healthy cattle was analyzed, and the key modules related to BRD were obtained based on WGCNA analysis (Figure 2, Figure 4, Additional table 2,4). Then, five potential biomarkers (ADGRG3, CDKN1A, CA4, GGT5, and SLC26A8) associated with BRD were identified through LASSO analysis and RF analysis, and the expression level of potential biomarkers was analyzed using dataset verification and RT-qPCR detection. Studies have shown that ADGRG3 is highly expressed in eosinophils, neutrophils and mast cells, which participates in macrophage inflammation induced by high-fat diet in obese mice and plays a key role in the occurrence and development of asthma [64-67]. Correlation studies of ADGRs reveales that ADGRG3, ADGRA1, ADGRF1, CD4T cells and CD8 cells were involved in the tumor-related inflammatory response of Uterine Corpus Endometrial Carcinoma (UCEC) patients, and affected the clinical prognosis of UCEC patients [68]. CDKN1A is involved in the regulation of cell replication, senescence, apoptosis and other processes, and is highly expressed in activated mast cells, eosinophils, neutrophils and memory CD4T cells [69, 70]. In nodular granuloma, high expression of CDKN1A leads to reduced apoptosis and persistent inflammation [71]. In addition, in glial cells induced by lipopolysaccharide (LPS), targeted interference with the expression of CDKN1A is related to the decreased activity of NF-kappaB [72]. Similarly, knockout of CDKN1A expression reduced lung inflammation in mice induced by smoking, LPS and N-formyl-methionyl-leucyl-phenylalanine (fMLP) [73]. CA4 is a zinc metalloenzyme involved in maintaining the dynamic balance of carbon dioxide and bicarbonate [74]. Asthma is a chronic inflammation characterized by eosinophil proliferation and cell activation. Studies have shown that eosinophils express CA4 after exposure to IL-5 or allergens, and CA4 is involved in the regulation of lung transcriptional groups associated with allergic respiratory inflammation [75]. GGT5 is a kind of cell surface protein, which is widely expressed in tissues and is mainly involved in biological processes such as inflammation, angiogenesis and immune response [76, 77]. In the study of the expression of GGT5 in gastric cancer and its correlation with immune cell infiltration, it was found that GGT5 was highly expressed in gastric cancer, and its expression level was positively correlated with the infiltration of dendritic cells, macrophages and natural killer cells, and negatively correlated with the infiltration of Th17 [77]. Screening of disease markers for colitis in mice using a machine-learning approach revealed that the expression levels of SLC26A8, MMP9, PTGDS, and CD160 were significantly elevated in colitis tissues, whereas the expression level of TLR5 was significantly reduced [78]. Sepsis is a life-threatening systemic inflammatory response, and septic shock is the most serious complication of sepsis [79, 80]. The marker genes of septic shock in children and their relationship with immune cells were studied by bioinformatics methods. SLC26A8, S100A9, KIF1B, S100A12 and UPP1 were identified as the early diagnostic marker genes of septic shock in children, and these marker genes may be involved in the infiltration of immune cells [81].

In this study, the signal transduction pathways related to potential biomarkers were analyzed by GSEA, and it was found that the genes positively related to potential biomarkers were mainly enriched in NOD-like receptor signaling pathway, Neutrophil extracellular trap formation, Necroptosis, Complement and coagulation cascades, T cell receptor signaling pathway, B cell receptor signaling pathway, Lysosome, Fc gamma R-mediated phagocytosis, Th1 and Th2 cell differentiation and IL-17 signaling pathway (Figure 7, Additional table 8). When the organism is invaded by pathogens, NOD-like receptor signaling pathway is activated and produces innate immune response, which in turn drives the activation of NF-κB and MAPK, the production of cytokines and apoptosis [82]. Neutrophil Extracellular Traps (NETs) is a reticular structure composed of DNA histone complexes and proteins released by activated neutrophils, which can capture viruses, bacteria and fungi and play a key role in neutrophil innate immune response and non-infectious diseases [83-86]. T cells play an important role in the adaptive immune system against foreign invasion, with T cell receptor (TCR) signaling being essential for their development and function [87, 88]. B cells can bind specific antigens through their B cell receptors (BCR) and present these antigens to T cells, which in turn triggers T cell immune response [89]. Necroptosis is accompanied by the release of damage-associated molecular model (DAMP) and cytokines, which triggers the pro-inflammatory response, which is a backup cellular defense mechanism [90]. Dysfunctional Necroptosis can lead to neuroinflammation, chronic intestinal inflammation, and inflammatory skin diseases [91-95]. Th2 cells promote the release of IL-13, IL-4 and IL-5 and mediate humoral immunity, while Th1 cells activated by IL-12 secrete IFN-γ and mediate cellular immunity. When the imbalance between Th1 cells and Th2 cells is considered to be the immunological basis of allergic rhinitis [96]. IL-17 is a landmark cytokine secreted by Th17 cells, which is necessary for the body to defend against extracellular fungal and bacterial infections, and is also one of the pathogenesis of many autoimmune inflammatory diseases [97]. The above studies show that the five potential biomarkers identified in this study play an active role in chronic inflammation and persistent inflammation.

BRD, also known as "transport fever", s believed to immunosuppress calveds due to various factors during transportation, which allows the respiratory tract to be invaded by numerous of foreign pathogens. In this study, bioinformatics algorithm was used to analyze the correlation between immune cell infiltration and characteristic genes in cattle with BRD and healthy cattle. The results showed that compared with healthy cattle, the expression of B cells naive, Monocytes and Neutrophils in cattle with BRD increased, while the expression of B cells memory, T cells CD8, T cells CD4 naive, T cells regulatory (Tregs) and NK cells resting decreased (Figure 8). Neutrophils are highly phagocytes and are one of the first cell types to be recruited to the site of infection. they play an important role in protecting the host from bacterial infection, eliminating pathogens and tissue remodeling [98]. Neutrophils are associated with the pathogenesis of many inflammatory diseases, especially respiratory diseases [99]. Studies suggest that neutrophils may contribute to excessive inflammation and tissue damage observed in cattle with BRD induced by transport stress, coexisting with disease and injury in the respiratory tract [100]. In addition, the researchers analyzed the effect of transport on calf peripheral blood lymphocyte subsets by flow cytometry and found that the percentage of all T lymphocyte subsets decreased significantly immediately after transport [101]. Meanwhile, the BRD related potential biomarkers identified in this study was positively correlated with the levels of B cells naive, Neutrophils, Monocytes and T cells CD4 memory activated, and negatively correlated with the expression of NK cells resting, T cells regulatory (Tregs), T cells CD4 naive, T cells CD8 and B cells memory (Figure 9). Studies have shown that CD4T cells are considered necessary for the elimination of BHV-1 virus, and CD4T cells and CD8T cells play a key role in the immune response to BRSV virus infection. In general, these results show that the animal's own immune response plays an important role in determining the susceptibility and severity of BRD, and the evaluation of blood immune parameters is very important for early detection of BRD.

5. Conclusions

In this study, we employed bioinformatics analysis and machine learning algorithms to identify the ADGRG3, CDKN1A, CA4, GGT5, and SLC26A8 as potential biomarkers for the diagnosis and treating BRD. We also found that the expression of these biomarkers was closely correlated with the levels of various infiltrating immune cells. The results of this study explain the pathogenesis of BRD from the perspective of immune infiltrating, which helps to better understand the immune response of BRD and provide reference for the early diagnosis and targeted drug research of BRD.

**CRediT authorship contribution statement:** H.S. and J.Z. conceived the idea and designed the research. H.S. and J.Z. wrote the original manuscript. H.S. and J.Z. performed all analyses. H.S., J.Z., X.S., L.Z., D.Y., P.Z., Y.L., J.Z., X.G., and X.Z. reviewed and edited the manuscript. H.S., X.Z. and X.G. provided financial support. All authors read and approved the final manuscript.

**Funding:** This work was supported by the earmarked fund for Innovation 2030-Major Project of Agricultural Biotech Breeding (2022ZD04013); Tianjin Key R&D Plan (23YFZCSN00170); Natural Science Foundation of Tianjin (23JCYBJC00260); Tianjin Academy of Agricultural Sciences Seed Industry Innovation Project (2024ZYCX012); Tianjin Science and Technology Plan Project (23ZYCGSN00090, 23ZYCGSN00110, 22ZXZYSN00010, 24YDTPJC00290, 24YDTPJC00480); Open project of State Key Laboratory of Animal Biotech Breeding (2024SKLAB6-4; 2024SKLAB6-11). The funding bodies played no role in the design of the study, collection, analysis, and interpretation of data and writing the manuscript.

**Author statement:** We declare that this manuscript is original, has not been published before, and is not currently being considered for publication elsewhere.

**Availability of data and materials:**

The datasets analyzed during the current study are available in public database; Cattle [GEO database, GSE162156; https://www.ncbi.nlm.nih.gov/geo/query/acc.cgi?acc=GSE162156; GSE150706; https://www.ncbi.nlm.nih.gov/geo/query/acc.cgi?acc=GSE150706].

**Declarations:**

Ethics approval and consent to participate: Not applicable.

Consent for publication:Not applicable.

Competing interests:The authors declare that they have no competing interests.

**Supplementary material:**

Additional figure 1: Data quality control of GSE162456 dataset. A. Inter-sample PCA analysis. B. RPKM expression pattern. C. RPKM density distribution. D. Correlation analysis between samples. E. Cluster analysis between samples.

Additional figure 2: Data quality control of GSE50706 dataset. A. Inter-sample PCA analysis. B. RPKM expression pattern. C. RPKM density distribution. D. Correlation analysis between samples. E. Cluster analysis between samples.

Additional figure 2: ROC analysis results of key genes. A. Analysis results of GSE162456 dataset. B.Analysis results of GSE150706 dataset.

Additional table 1：Raw data from GSE162456 dataset.

Additional table 2：Difference analysis results.

Additional table 3：GO and KEGG analysis results.

Additional table 4：WGCNA analysis results.

Additional table 5：LASSO algorithm analysis results.

Additional table 6：RF algorithm analysis results.

Additional table 7：Raw data from GSE150706 dataset.

Additional table 8：GSEA analysis results of key genes.

Additional table 9：CIBERSORT algorithm analysis results.

Additional table 10：Primer information for RT-qPCR to detect gene expression.

References

1. Joshi V, Gupta VK, Bhanuprakash AG, Mandal RSK, Dimri U, Ajith Y: Haptoglobin and serum amyloid A as putative biomarker candidates of naturally occurring bovine respiratory disease in dairy calves. Microb Pathog. 2018; 116:33-37. doi:10.1016/j.micpath.2018.01.001.

2. Autio T, Pohjanvirta T, Holopainen R, Rikula U, Pentikainen J, Huovilainen A, Rusanen H, Soveri T, Sihvonen L, Pelkonen S: Etiology of respiratory disease in non-vaccinated, non-medicated calves in rearing herds. Vet Microbiol. 2007; 119(2-4):256-265. doi:10.1016/j.vetmic.2006.10.001.

3. Orro T, Pohjanvirta T, Rikula U, Huovilainen A, Alasuutari S, Sihvonen L, Pelkonen S, Soveri T: Acute phase protein changes in calves during an outbreak of respiratory disease caused by bovine respiratory syncytial virus. Comp Immunol Microbiol Infect Dis. 2011; 34(1):23-29. doi:10.1016/j.cimid.2009.10.005.

4. Dubrovsky SA, Van Eenennaam AL, Aly SS, Karle BM, Rossitto PV, Overton MW, Lehenbauer TW, Fadel JG: Preweaning cost of bovine respiratory disease (BRD) and cost-benefit of implementation of preventative measures in calves on California dairies: The BRD 10K study. J Dairy Sci. 2020; 103(2):1583-1597. doi:10.3168/jds.2018-15501.

5. Gorden PJ, Plummer P: Control, management, and prevention of bovine respiratory disease in dairy calves and cows. Vet Clin North Am Food Anim Pract. 2010; 26(2):243-259. doi:10.1016/j.cvfa.2010.03.004.

6. Santos-Rivera M, Fitzkee NC, Hill RA, Baird RE, Blair E, Thoresen M, Woolums AR, Meyer F, Vance CK: NMR-based metabolomics of plasma from dairy calves infected with two primary causal agents of bovine respiratory disease (BRD). Sci Rep. 2023; 13(1):2671. doi:10.1038/s41598-023-29234-3.

7. Galyean ML, Duff GC, Rivera JD: Galyean Appreciation Club Review: revisiting nutrition and health of newly received cattle-what have we learned in the last 15 years? J Anim Sci. 2022; 100(4). doi:10.1093/jas/skac067.

8. Dubrovsky SA, Van Eenennaam AL, Karle BM, Rossitto PV, Lehenbauer TW, Aly SS: Epidemiology of bovine respiratory disease (BRD) in preweaned calves on California dairies: The BRD 10K study. J Dairy Sci. 2019; 102(8):7306-7319. doi:10.3168/jds.2018-14774.

9. Bull EM, Bartram DJ, Cock B, Odeyemi I, Main DCJ: Construction of a conceptual framework for assessment of health-related quality of life in calves with respiratory disease. Animal. 2021; 15(4):100191. doi:10.1016/j.animal.2021.100191.

10. Holland BP, Burciaga-Robles LO, VanOverbeke DL, Shook JN, Step DL, Richards CJ, Krehbiel CR: Effect of bovine respiratory disease during preconditioning on subsequent feedlot performance, carcass characteristics, and beef attributes. J Anim Sci. 2010; 88(7):2486-2499. doi:10.2527/jas.2009-2428.

11. Wilson BK, Step DL, Maxwell CL, Gifford CA, Richards CJ, Krehbiel CR: Effect of bovine respiratory disease during the receiving period on steer finishing performance, efficiency, carcass characteristics, and lung scores. Prof Anim Sci. 2017; 33(1):24-36. doi:10.15232/pas.2016-01554.

12. Blakebrough-Hall C, McMeniman JP, Gonzalez LA: An evaluation of the economic effects of bovine respiratory disease on animal performance, carcass traits, and economic outcomes in feedlot cattle defined using four BRD diagnosis methods. J Anim Sci. 2020; 98(2). doi:10.1093/jas/skaa005.

13. Stanton AL, Kelton DF, LeBlanc SJ, Wormuth J, Leslie KE: The effect of respiratory disease and a preventative antibiotic treatment on growth, survival, age at first calving, and milk production of dairy heifers. J Dairy Sci. 2012; 95(9):4950-4960. doi:10.3168/jds.2011-5067.

14. Blakebrough-Hall C, Dona A, D'Occhio M J, McMeniman J, Gonzalez LA: Diagnosis of Bovine Respiratory Disease in feedlot cattle using blood (1)H NMR metabolomics. Sci Rep. 2020; 10(1):115. doi:10.1038/s41598-019-56809-w.

15. Schaffer AP, Larson RL, Cernicchiaro N, Hanzlicek GA, Bartle SJ, Thomson DU: The association between calfhood bovine respiratory disease complex and subsequent departure from the herd, milk production, and reproduction in dairy cattle. J Am Vet Med Assoc. 2016; 248(10):1157-1164. doi:10.2460/javma.248.10.1157.

16. Bach A: Associations between several aspects of heifer development and dairy cow survivability to second lactation. J Dairy Sci. 2011; 94(2):1052-1057. doi:10.3168/jds.2010-3633.

17. Wilson BK, Richards CJ, Step DL, Krehbiel CR: Best management practices for newly weaned calves for improved health and well-being. J Anim Sci. 2017; 95(5):2170-2182. doi:10.2527/jas.2016.1006.

18. Porter MM, McDonald PO, Slate JR, Kreuder AJ, McGill JL: Use of Thoracic Ultrasonography to Improve Disease Detection in Experimental BRD Infection. Front Vet Sci. 2021; 8:763972. doi:10.3389/fvets.2021.763972.

19. McGuirk SM, Peek SF: Timely diagnosis of dairy calf respiratory disease using a standardized scoring system. Anim Health Res Rev. 2014; 15(2):145-147. doi:10.1017/S1466252314000267.

20. Buczinski S, Forte G, Francoz D, Belanger AM: Comparison of thoracic auscultation, clinical score, and ultrasonography as indicators of bovine respiratory disease in preweaned dairy calves. J Vet Intern Med. 2014; 28(1):234-242. doi:10.1111/jvim.12251.

21. Timsit E, Dendukuri N, Schiller I, Buczinski S: Diagnostic accuracy of clinical illness for bovine respiratory disease (BRD) diagnosis in beef cattle placed in feedlots: A systematic literature review and hierarchical Bayesian latent-class meta-analysis. Prev Vet Med. 2016; 135:67-73. doi:10.1016/j.prevetmed.2016.11.006.

22. Buczinski S, Faure C, Jolivet S, Abdallah A: Evaluation of inter-observer agreement when using a clinical respiratory scoring system in pre-weaned dairy calves. N Z Vet J. 2016; 64(4):243-247. doi:10.1080/00480169.2016.1153439.

23. Berman J, Francoz D, Abdallah A, Dufour S, Buczinski S: Evaluation of inter-rater agreement of the clinical signs used to diagnose bovine respiratory disease in individually housed veal calves. J Dairy Sci. 2021; 104(11):12053-12065. doi:10.3168/jds.2021-20503.

24. Maier GU, Rowe JD, Lehenbauer TW, Karle BM, Williams DR, Champagne JD, Aly SS: Development of a clinical scoring system for bovine respiratory disease in weaned dairy calves. J Dairy Sci. 2019; 102(8):7329-7344. doi:10.3168/jds.2018-15474.

25. Love WJ, Lehenbauer TW, Van Eenennaam AL, Drake CM, Kass PH, Farver TB, Aly SS: Sensitivity and specificity of on-farm scoring systems and nasal culture to detect bovine respiratory disease complex in preweaned dairy calves. J Vet Diagn Invest. 2016; 28(2):119-128. doi:10.1177/1040638715626204.

26. Buczinski S, T LO, Dendukuri N: Bayesian estimation of the accuracy of the calf respiratory scoring chart and ultrasonography for the diagnosis of bovine respiratory disease in pre-weaned dairy calves. Prev Vet Med. 2015; 119(3-4):227-231. doi:10.1016/j.prevetmed.2015.02.018.

27. Jiminez J, Timsit E, Orsel K, van der Meer F, Guan LL, Plastow G: Whole-Blood Transcriptome Analysis of Feedlot Cattle With and Without Bovine Respiratory Disease. Front Genet. 2021; 12:627623. doi:10.3389/fgene.2021.627623.

28. Ling Y, Li J, Zhou L: Smoking-related epigenetic modifications are associated with the prognosis and chemotherapeutics of patients with bladder cancer. Int J Immunopathol Pharmacol. 2023; 37:3946320231166774. doi:10.1177/03946320231166774.

29. Yang H, Huo P, Hu G, Wei B, Kong D, Li H: Identification of gene markers associated with metastasis in clear cell renal cell carcinoma. Oncol Lett. 2017; 13(6):4755-4761. doi:10.3892/ol.2017.6084.

30. Ito K, Murphy D: Application of ggplot2 to Pharmacometric Graphics. CPT Pharmacometrics Syst Pharmacol. 2013; 2(10):e79. doi:10.1038/psp.2013.56.

31. Zhao Z, Yang H, Ji G, Su S, Fan Y, Wang M, Gu S: Identification of hub genes for early detection of bone metastasis in breast cancer. Front Endocrinol (Lausanne). 2022; 13:1018639. doi:10.3389/fendo.2022.1018639.

32. Tang S, Jing H, Huang Z, Huang T, Lin S, Liao M, Zhou J: Identification of key candidate genes in neuropathic pain by integrated bioinformatic analysis. J Cell Biochem. 2020; 121(2):1635-1648. doi:10.1002/jcb.29398.

33. Li GM, Zhang CL, Rui RP, Sun B, Guo W: Bioinformatics analysis of common differential genes of coronary artery disease and ischemic cardiomyopathy. Eur Rev Med Pharmacol Sci. 2018; 22(11):3553-3569. doi:10.26355/eurrev_201806_15182.

34. Kazerani R, Salehipour P, Shah Mohammadi M, Amanzadeh Jajin E, Modarressi MH: Identification of TSGA10 and GGNBP2 splicing variants in 5' untranslated region with distinct expression profiles in brain tumor samples. Front Oncol. 2023; 13:1075638. doi:10.3389/fonc.2023.1075638.

35. Yu G, Wang LG, Han Y, He QY: clusterProfiler: an R package for comparing biological themes among gene clusters. Omics. 2012; 16(5):284-287. doi:10.1089/omi.2011.0118.

36. Wu T, Hu E, Xu S, Chen M, Guo P, Dai Z, Feng T, Zhou L, Tang W, Zhan L *et al*: clusterProfiler 4.0: A universal enrichment tool for interpreting omics data. Innovation (Camb). 2021; 2(3):100141. doi:10.1016/j.xinn.2021.100141.

37. Langfelder P, Horvath S: WGCNA: an R package for weighted correlation network analysis. BMC Bioinformatics. 2008; 9:559. doi:10.1186/1471-2105-9-559.

38. Serra A, Onlu S, Coretto P, Greco D: An integrated quantitative structure and mechanism of action-activity relationship model of human serum albumin binding. J Cheminform. 2019; 11(1):38. doi:10.1186/s13321-019-0359-2.

39. Wang J, Shi L: Prediction of medical expenditures of diagnosed diabetics and the assessment of its related factors using a random forest model, MEPS 2000-2015. Int J Qual Health Care. 2020; 32(2):99-112. doi:10.1093/intqhc/mzz135.

40. Zhang M, Zhu K, Pu H, Wang Z, Zhao H, Zhang J, Wang Y: An Immune-Related Signature Predicts Survival in Patients With Lung Adenocarcinoma. Front Oncol. 2019; 9:1314. doi:10.3389/fonc.2019.01314.

41. Alderden J, Pepper GA, Wilson A, Whitney JD, Richardson S, Butcher R, Jo Y, Cummins MR: Predicting Pressure Injury in Critical Care Patients: A Machine-Learning Model. Am J Crit Care. 2018; 27(6):461-468. doi:10.4037/ajcc2018525.

42. Robin X, Turck N, Hainard A, Tiberti N, Lisacek F, Sanchez JC, Muller M: pROC: an open-source package for R and S+ to analyze and compare ROC curves. BMC Bioinformatics. 2011; 12:77. doi:10.1186/1471-2105-12-77.

43. Mandrekar JN: Receiver operating characteristic curve in diagnostic test assessment. J Thorac Oncol. 2010; 5(9):1315-1316. doi:10.1097/JTO.0b013e3181ec173d.

44. Ilhan ZE, Laniewski P, Thomas N, Roe DJ, Chase DM, Herbst-Kralovetz MM: Deciphering the complex interplay between microbiota, HPV, inflammation and cancer through cervicovaginal metabolic profiling. EBioMedicine. 2019; 44:675-690. doi:10.1016/j.ebiom.2019.04.028.

45. Cooper JD, Han SYS, Tomasik J, Ozcan S, Rustogi N, van Beveren NJM, Leweke FM, Bahn S: Multimodel inference for biomarker development: an application to schizophrenia. Transl Psychiatry. 2019; 9(1):83. doi:10.1038/s41398-019-0419-4.

46. Paraskevaidi M, Morais CLM, Ashton KM, Stringfellow HF, McVey RJ, Ryan NAJ, O'Flynn H, Sivalingam VN, Kitson SJ, MacKintosh ML *et al*: Detecting Endometrial Cancer by Blood Spectroscopy: A Diagnostic Cross-Sectional Study. Cancers (Basel). 2020; 12(5). doi:10.3390/cancers12051256.

47. Powers RK, Goodspeed A, Pielke-Lombardo H, Tan AC, Costello JC: GSEA-InContext: identifying novel and common patterns in expression experiments. Bioinformatics. 2018; 34(13):i555-i564. doi:10.1093/bioinformatics/bty271.

48. Newman AM, Liu CL, Green MR, Gentles AJ, Feng W, Xu Y, Hoang CD, Diehn M, Alizadeh AA: Robust enumeration of cell subsets from tissue expression profiles. Nat Methods. 2015; 12(5):453-457. doi:10.1038/nmeth.3337.

49. Newman AM, Steen CB, Liu CL, Gentles AJ, Chaudhuri AA, Scherer F, Khodadoust MS, Esfahani MS, Luca BA, Steiner D *et al*: Determining cell type abundance and expression from bulk tissues with digital cytometry. Nat Biotechnol. 2019; 37(7):773-782. doi:10.1038/s41587-019-0114-2.

50. Huang R, Zheng X, Wang J: Bioinformatic exploration of the immune related molecular mechanism underlying pulmonary arterial hypertension. Bioengineered. 2021; 12(1):3137-3147. doi:10.1080/21655979.2021.1944720.

51. Sheng H, Pan C, Wang S, Yang C, Zhang J, Hu C, Hu H, Feng X, Yang M, Lei Z *et al*: Weighted Gene Co-Expression Network Analysis Identifies Key Modules and Central Genes Associated With Bovine Subcutaneous Adipose Tissue. Front Vet Sci. 2022; 9:914848. doi:10.3389/fvets.2022.914848.

52. Zhang J, Sheng H, Pan C, Wang S, Yang M, Hu C, Wei D, Wang Y, Ma Y: Identification of key genes in bovine muscle development by co-expression analysis. PeerJ. 2023; 11:e15093. doi:10.7717/peerj.15093.

53. Cheng Q, Chen X, Wu H, Du Y: Three hematologic/immune system-specific expressed genes are considered as the potential biomarkers for the diagnosis of early rheumatoid arthritis through bioinformatics analysis. J Transl Med. 2021; 19(1):18. doi:10.1186/s12967-020-02689-y.

54. Bochukova EG, Lawler K, Croizier S, Keogh JM, Patel N, Strohbehn G, Lo KK, Humphrey J, Hokken-Koelega A, Damen L *et al*: A Transcriptomic Signature of the Hypothalamic Response to Fasting and BDNF Deficiency in Prader-Willi Syndrome. Cell Rep. 2018; 22(13):3401-3408. doi:10.1016/j.celrep.2018.03.018.

55. Smith RA, Step DL, Woolums AR: Bovine Respiratory Disease: Looking Back and Looking Forward, What Do We See? Vet Clin North Am Food Anim Pract. 2020; 36(2):239-251. doi:10.1016/j.cvfa.2020.03.009.

56. Scott MA, Woolums AR, Swiderski CE, Perkins AD, Nanduri B, Smith DR, Karisch BB, Epperson WB, Blanton JR, Jr.: Whole blood transcriptomic analysis of beef cattle at arrival identifies potential predictive molecules and mechanisms that indicate animals that naturally resist bovine respiratory disease. PLoS One. 2020; 15(1):e0227507. doi:10.1371/journal.pone.0227507.

57. Scott MA, Woolums AR, Swiderski CE, Perkins AD, Nanduri B, Smith DR, Karisch BB, Epperson WB, Blanton JR: Comprehensive at-arrival transcriptomic analysis of post-weaned beef cattle uncovers type I interferon and antiviral mechanisms associated with bovine respiratory disease mortality. PLoS One. 2021; 16(4):e0250758. doi:10.1371/journal.pone.0250758.

58. Johnston D, Earley B, McCabe MS, Kim J, Taylor JF, Lemon K, Duffy C, McMenamy M, Cosby SL, Waters SM: Messenger RNA biomarkers of Bovine Respiratory Syncytial Virus infection in the whole blood of dairy calves. Sci Rep. 2021; 11(1):9392. doi:10.1038/s41598-021-88878-1.

59. Lindholm-Perry AK, Kuehn LA, McDaneld TG, Miles JR, Workman AM, Chitko-McKown CG, Keele JW: Complete blood count data and leukocyte expression of cytokine genes and cytokine receptor genes associated with bovine respiratory disease in calves. BMC Res Notes. 2018; 11(1):786. doi:10.1186/s13104-018-3900-x.

60. Tizioto PC, Kim J, Seabury CM, Schnabel RD, Gershwin LJ, Van Eenennaam AL, Toaff-Rosenstein R, Neibergs HL, Bovine Respiratory Disease Complex Coordinated Agricultural Project Research T, Taylor JF: Immunological Response to Single Pathogen Challenge with Agents of the Bovine Respiratory Disease Complex: An RNA-Sequence Analysis of the Bronchial Lymph Node Transcriptome. PLoS One. 2015; 10(6):e0131459. doi:10.1371/journal.pone.0131459.

61. Johnston D, Earley B, McCabe MS, Lemon K, Duffy C, McMenamy M, Cosby SL, Kim J, Blackshields G, Taylor JF *et al*: Experimental challenge with bovine respiratory syncytial virus in dairy calves: bronchial lymph node transcriptome response. Sci Rep. 2019; 9(1):14736. doi:10.1038/s41598-019-51094-z.

62. Sun HZ, Srithayakumar V, Jiminez J, Jin W, Hosseini A, Raszek M, Orsel K, Guan LL, Plastow G: Longitudinal blood transcriptomic analysis to identify molecular regulatory patterns of bovine respiratory disease in beef cattle. Genomics. 2020; 112(6):3968-3977. doi:10.1016/j.ygeno.2020.07.014.

63. Mukherjee K, Edgett BA, Burrows HW, Castro C, Griffin JL, Schwertani AG, Gurd BJ, Funk CD: Whole blood transcriptomics and urinary metabolomics to define adaptive biochemical pathways of high-intensity exercise in 50-60 year old masters athletes. PLoS One. 2014; 9(3):e92031. doi:10.1371/journal.pone.0092031.

64. Peng YM, van de Garde MD, Cheng KF, Baars PA, Remmerswaal EB, van Lier RA, Mackay CR, Lin HH, Hamann J: Specific expression of GPR56 by human cytotoxic lymphocytes. J Leukoc Biol. 2011; 90(4):735-740. doi:10.1189/jlb.0211092.

65. Valtcheva N, Primorac A, Jurisic G, Hollmen M, Detmar M: The orphan adhesion G protein-coupled receptor GPR97 regulates migration of lymphatic endothelial cells via the small GTPases RhoA and Cdc42. J Biol Chem. 2013; 288(50):35736-35748. doi:10.1074/jbc.M113.512954.

66. Shi J, Zhang X, Wang S, Wang J, Du B, Wang Z, Liu M, Jiang W, Qian M, Ren H: Gpr97 is dispensable for metabolic syndrome but is involved in macrophage inflammation in high-fat diet-induced obesity in mice. Sci Rep. 2016; 6:24649. doi:10.1038/srep24649.

67. Shi JP, Li XN, Zhang XY, Du B, Jiang WZ, Liu MY, Wang JJ, Wang ZG, Ren H, Qian M: Gpr97 Is Dispensable for Inflammation in OVA-Induced Asthmatic Mice. PLoS One. 2015; 10(7):e0131461. doi:10.1371/journal.pone.0131461.

68. Lei P, Wang H, Yu L, Xu C, Sun H, Lyu Y, Li L, Zhang DL: A correlation study of adhesion G protein-coupled receptors as potential therapeutic targets in Uterine Corpus Endometrial cancer. Int Immunopharmacol. 2022; 108:108743. doi:10.1016/j.intimp.2022.108743.

69. Santiago-Raber ML, Lawson BR, Dummer W, Barnhouse M, Koundouris S, Wilson CB, Kono DH, Theofilopoulos AN: Role of cyclin kinase inhibitor p21 in systemic autoimmunity. J Immunol. 2001; 167(7):4067-4074. doi:10.4049/jimmunol.167.7.4067.

70. Chen H, Liu J, Wu Y, Jiang L, Tang M, Wang X, Fang X, Wang X: Weighted gene co-expression identification of CDKN1A as a hub inflammation gene following cardiopulmonary bypass in children with congenital heart disease. Front Surg. 2022; 9:963850. doi:10.3389/fsurg.2022.963850.

71. Xaus J, Besalduch N, Comalada M, Marcoval J, Pujol R, Mana J, Celada A: High expression of p21 Waf1 in sarcoid granulomas: a putative role for long-lasting inflammation. J Leukoc Biol. 2003; 74(2):295-301. doi:10.1189/jlb.1202628.

72. Tusell JM, Saura J, Serratosa J: Absence of the cell cycle inhibitor p21Cip1 reduces LPS-induced NO release and activation of the transcription factor NF-kappaB in mixed glial cultures. Glia. 2005; 49(1):52-58. doi:10.1002/glia.20095.

73. Yao H, Yang SR, Edirisinghe I, Rajendrasozhan S, Caito S, Adenuga D, O'Reilly MA, Rahman I: Disruption of p21 attenuates lung inflammation induced by cigarette smoke, LPS, and fMLP in mice. Am J Respir Cell Mol Biol. 2008; 39(1):7-18. doi:10.1165/rcmb.2007-0342OC.

74. Xu Y, Xu WH, Shi SN, Yang XL, Ren YR, Zhuang XY, Qu YY, Zhang HL, Zhang XF: Carbonic Anhydrase 4 serves as a Clinicopathological Biomarker for Outcomes and Immune Infiltration in Renal Cell Carcinoma, Lower Grade Glioma, Lung Adenocarcinoma and Uveal Melanoma. J Cancer. 2020; 11(20):6101-6113. doi:10.7150/jca.46902.

75. Wen T, Mingler MK, Wahl B, Khorki ME, Pabst O, Zimmermann N, Rothenberg ME: Carbonic anhydrase IV is expressed on IL-5-activated murine eosinophils. J Immunol. 2014; 192(12):5481-5489. doi:10.4049/jimmunol.1302846.

76. Hanigan MH, Gillies EM, Wickham S, Wakeham N, Wirsig-Wiechmann CR: Immunolabeling of gamma-glutamyl transferase 5 in normal human tissues reveals that expression and localization differ from gamma-glutamyl transferase 1. Histochem Cell Biol. 2015; 143(5):505-515. doi:10.1007/s00418-014-1295-x.

77. Wang Y, Fang Y, Zhao F, Gu J, Lv X, Xu R, Zhang B, Fang Z, Li Y: Identification of GGT5 as a Novel Prognostic Biomarker for Gastric Cancer and its Correlation With Immune Cell Infiltration. Front Genet. 2022; 13:810292. doi:10.3389/fgene.2022.810292.

78. Mei K, Chen Z, Wang Q, Luo Y, Huang Y, Wang B, Gu R: The role of intestinal immune cells and matrix metalloproteinases in inflammatory bowel disease. Front Immunol. 2022; 13:1067950. doi:10.3389/fimmu.2022.1067950.

79. Evans L, Rhodes A, Alhazzani W, Antonelli M, Coopersmith CM, French C, Machado FR, McIntyre L, Ostermann M, Prescott HC *et al*: Executive Summary: Surviving Sepsis Campaign: International Guidelines for the Management of Sepsis and Septic Shock 2021. Crit Care Med. 2021; 49(11):1974-1982. doi:10.1097/CCM.0000000000005357.

80. Garcia PCR, Tonial CT, Piva JP: Septic shock in pediatrics: the state-of-the-art. J Pediatr (Rio J). 2020; 96 Suppl 1(Suppl 1):87-98. doi:10.1016/j.jped.2019.10.007.

81. Fan J, Shi S, Qiu Y, Liu M, Shu Q: Analysis of signature genes and association with immune cells infiltration in pediatric septic shock. Front Immunol. 2022; 13:1056750. doi:10.3389/fimmu.2022.1056750.

82. Qiu D, Zhang D, Yu Z, Jiang Y, Zhu D: Bioinformatics approach reveals the critical role of the NOD-like receptor signaling pathway in COVID-19-associated multiple sclerosis syndrome. J Neural Transm (Vienna). 2022; 129(8):1031-1038. doi:10.1007/s00702-022-02518-0.

83. Brinkmann V, Reichard U, Goosmann C, Fauler B, Uhlemann Y, Weiss DS, Weinrauch Y, Zychlinsky A: Neutrophil extracellular traps kill bacteria. Science. 2004; 303(5663):1532-1535. doi:10.1126/science.1092385.

84. Dabrowska D, Jablonska E, Garley M, Ratajczak-Wrona W, Iwaniuk A: New Aspects of the Biology of Neutrophil Extracellular Traps. Scand J Immunol. 2016; 84(6):317-322. doi:10.1111/sji.12494.

85. Berger-Achituv S, Brinkmann V, Abed UA, Kuhn LI, Ben-Ezra J, Elhasid R, Zychlinsky A: A proposed role for neutrophil extracellular traps in cancer immunoediting. Front Immunol. 2013; 4:48. doi:10.3389/fimmu.2013.00048.

86. Brinkmann V: Neutrophil Extracellular Traps in the Second Decade. J Innate Immun. 2018; 10(5-6):414-421. doi:10.1159/000489829.

87. Kim S, Park GY, Park JS, Park J, Hong H, Lee Y: Regulation of positive and negative selection and TCR signaling during thymic T cell development by capicua. Elife. 2021; 10. doi:10.7554/eLife.71769.

88. Takeuchi Y, Hirota K, Sakaguchi S: Impaired T cell receptor signaling and development of T cell-mediated autoimmune arthritis. Immunol Rev. 2020; 294(1):164-176. doi:10.1111/imr.12841.

89. Puri KD, Di Paolo JA, Gold MR: B-cell receptor signaling inhibitors for treatment of autoimmune inflammatory diseases and B-cell malignancies. Int Rev Immunol. 2013; 32(4):397-427. doi:10.3109/08830185.2013.818140.

90. Zhang T, Wang Y, Inuzuka H, Wei W: Necroptosis pathways in tumorigenesis. Semin Cancer Biol. 2022; 86(Pt 3):32-40. doi:10.1016/j.semcancer.2022.07.007.

91. Yuan J, Amin P, Ofengeim D: Necroptosis and RIPK1-mediated neuroinflammation in CNS diseases. Nat Rev Neurosci. 2019; 20(1):19-33. doi:10.1038/s41583-018-0093-1.

92. Welz PS, Wullaert A, Vlantis K, Kondylis V, Fernandez-Majada V, Ermolaeva M, Kirsch P, Sterner-Kock A, van Loo G, Pasparakis M: FADD prevents RIP3-mediated epithelial cell necrosis and chronic intestinal inflammation. Nature. 2011; 477(7364):330-334. doi:10.1038/nature10273.

93. Gunther C, Martini E, Wittkopf N, Amann K, Weigmann B, Neumann H, Waldner MJ, Hedrick SM, Tenzer S, Neurath MF *et al*: Caspase-8 regulates TNF-alpha-induced epithelial necroptosis and terminal ileitis. Nature. 2011; 477(7364):335-339. doi:10.1038/nature10400.

94. Dannappel M, Vlantis K, Kumari S, Polykratis A, Kim C, Wachsmuth L, Eftychi C, Lin J, Corona T, Hermance N *et al*: RIPK1 maintains epithelial homeostasis by inhibiting apoptosis and necroptosis. Nature. 2014; 513(7516):90-94. doi:10.1038/nature13608.

95. Bonnet MC, Preukschat D, Welz PS, van Loo G, Ermolaeva MA, Bloch W, Haase I, Pasparakis M: The adaptor protein FADD protects epidermal keratinocytes from necroptosis in vivo and prevents skin inflammation. Immunity. 2011; 35(4):572-582. doi:10.1016/j.immuni.2011.08.014.

96. Ke X, Chen Z, Wang X, Kang H, Hong S: Quercetin improves the imbalance of Th1/Th2 cells and Treg/Th17 cells to attenuate allergic rhinitis. Autoimmunity. 2023; 56(1):2189133. doi:10.1080/08916934.2023.2189133.

97. Qian Y, Kang Z, Liu C, Li X: IL-17 signaling in host defense and inflammatory diseases. Cell Mol Immunol. 2010; 7(5):328-333. doi:10.1038/cmi.2010.27.

98. McGill JL, Sacco RE: The Immunology of Bovine Respiratory Disease: Recent Advancements. Vet Clin North Am Food Anim Pract. 2020; 36(2):333-348. doi:10.1016/j.cvfa.2020.03.002.

99. Earley B, Buckham Sporer K, Gupta S: Invited review: Relationship between cattle transport, immunity and respiratory disease. Animal. 2017; 11(3):486-492. doi:10.1017/S1751731116001622.

100. Maheswaran SK, Weiss DJ, Kannan MS, Townsend EL, Reddy KR, Whiteley LO, Srikumaran S: Effects of Pasteurella haemolytica A1 leukotoxin on bovine neutrophils: degranulation and generation of oxygen-derived free radicals. Vet Immunol Immunopathol. 1992; 33(1-2):51-68. doi:10.1016/0165-2427(92)90034-n.

101. Riondato F, D'Angelo A, Miniscalco B, Bellino C, Guglielmino R: Effects of road transportation on lymphocyte subsets in calves. Vet J. 2008; 175(3):364-368. doi:10.1016/j.tvjl.2007.02.001.
