# Supplementary material for: Identification of diagnostic biomarkers of and immune cell infiltration analysis in bovine respiratory disease
Source: Front Vet Sci. 2025 Mar 5;12:1556676. doi: 10.3389/fvets.2025.1556676 (PMC11921050; doi:10.3389/fvets.2025.1556676)
Supplement: Supplementary file 15 [file Table_12.docx]

1.Quality Control

ls /data/sherry/*/*.gz |while read id;do(echo fastqc -t 5 $id);done >raw_qc.sh

for i in {0..19};do ( nohup bash /data/sherry/jimmy/submit.sh raw_qc.sh 20 $i 1>log_qc_$i.txt 2>&1 & ) ;done

ls /home/sherry/add/b93-PRJDA72405/*.gz |while read id;do(echo fastqc -t 5 $id);done >qc.sh

2.filter

#Double-ended

ls /data/sherry/*/*_1.fastq.gz|cut -d"_" -f 1|sort -u |while read id;do

echo trim_galore -q 25 --phred33 --length 36 --stringency 3 --paired -o /data/sherry/add/cleanData ${id}_*.gz

done > paired_trim_galore.sh

#Batch Run

for i in {0..19};do ( nohup bash /data/sherry/jimmy/submit.sh paired_trim_galore.sh 20 $i 1>log.batch1.paired_trim_galore.$i.txt 2>&1 & ) ;done

#Single-ended

ls /data/sherry/*/*gz|grep -v "_" |sort -u |while read id;do

echo trim_galore -q 25 --phred33 --length 36 --stringency 3 -o /data/sherry/add/1cleanData ${id}

done > single_trim_galore.sh

#Batch Run

for i in {0..19};do ( nohup bash /data/sherry/jimmy/submit.sh single_trim_galore.sh 20 $i 1>log.single_trim_galore.$i.txt 2>&1 & ) ;done

submit.sh

cat submit.sh

cat $1 |while read id

do

if((i%$2==$3))

then

$id

fi # check the number1 number2

i=$((i+1))

done

# for i in {0..9};do ( nohup bash ../submit.sh trim_galore.sh 10 $i 1>log.trim_galore.$i.txt 2>&1) ;done

3.Hisat2

#Building an index

nohup hisat2-build -p 8 Bos_taurus.ARS-UCD1.2.dna.toplevel.fa Bos_taurus.ARS-UCD1.2.dna.toplevel.hisat2.index &

ls -lh Bos_taurus.ARS-UCD1.2.dna.toplevel.hisat2.index*|cut -d" " -f 5-

868M May 17 22:42 Bos_taurus.ARS-UCD1.2.dna.toplevel.hisat2.index.1.ht2

648M May 17 22:42 Bos_taurus.ARS-UCD1.2.dna.toplevel.hisat2.index.2.ht2

23K May 17 22:25 Bos_taurus.ARS-UCD1.2.dna.toplevel.hisat2.index.3.ht2

648M May 17 22:25 Bos_taurus.ARS-UCD1.2.dna.toplevel.hisat2.index.4.ht2

1.2G May 17 22:46 Bos_taurus.ARS-UCD1.2.dna.toplevel.hisat2.index.5.ht2

660M May 17 22:46 Bos_taurus.ARS-UCD1.2.dna.toplevel.hisat2.index.6.ht2

12 May 17 22:26 Bos_taurus.ARS-UCD1.2.dna.toplevel.hisat2.index.7.ht2

8 May 17 22:26 Bos_taurus.ARS-UCD1.2.dna.toplevel.hisat2.index.8.ht2

## Quantitatively obtain TPM value

cd cleanData

mkdir hisat2_output

indexPrefix=/home/sherry/all-clean-data/ref/Bos_taurus.ARS-UCD1.2.dna.toplevel.hisat2.index

ls -lh $indexPrefix*

# double-ended sequencing

# run_hisat2_pe.sh

# ls *val*gz|cut -d"_" -f 1|sort -u |head

indexPrefix=/home/sherry/all-clean-data/ref/Bos_taurus.ARS-UCD1.2.dna.toplevel.hisat2.index

#ls -lh $indexPrefix*

ls *val*gz|cut -d"_" -f 1|sort -u |while read id;do

if((i%$1==$2))

then

hisat2 -p 2 -x $indexPrefix -1 ${id}*_1_val_1.fq.gz -2 ${id}*_2_val_2.fq.gz -S ${id}.hisat.sam

samtools sort -O bam -@ 2 -o ${id}.hisat.bam ${id}.hisat.sam

rm ${id}.hisat.sam

samtools index ${id}.hisat.bam

fi # check the number1 number2

i=$((i+1))

done

#run_hisat2_se.sh

# Single-end sequencing

indexPrefix=/home/sherry/all-clean-data/ref/Bos_taurus.ARS-UCD1.2.dna.toplevel.hisat2.index

ls -lh $indexPrefix*

ls *gz|grep -v "_1" |grep -v "_2" |sort -u |while read id;do

if((i%$1==$2))

then

hisat2 -p 2 -x $indexPrefix -U $id -S ${id%%_*}.hisat_se.sam

samtools sort -O bam -@ 2 -o ${id%%_*}.hisat_se.bam ${id%%_*}.hisat_se.sam

# rm ${id}.hisat_se.sam

samtools index ${id%%_*}.hisat_se.bam

fi # check the number1 number2

i=$((i+1))

done

#Batch Submission

for i in {0..19};do ( nohup bash run_hisat2_pe.sh 20 $i 1>log.hisat2.$i.txt 2>&1 & ) ;done

for i in {0..19};do ( nohup bash run_hisat2_se.sh 20 $i 1>log.hisat2_se.$i.txt 2>&1 & ) ;done

4.Perform qualimap quality control on bam files

#Double-ended

bin=/home/sherry/all-clean-data/software/qualimap_v2.2.1/qualimap

gtf=/home/sherry/index/Bos_taurus.ARS-UCD1.2.102.gtf

#pe_bam=/data/sherry/add/cleanData/paired_hisat2_output/

output=/home/sherry/add/cleanData/qualimap_pe_hisat2

ls *.bam|while read id;do

echo $bin rnaseq --java-mem-size=40G -bam $id -gtf $gtf -outdir $output/$(basename ${id} ".hisat.bam")_qc -outformat PDF:HTML -oc $(basename ${id} ".hisat.bam")

done > qualimap.sh

ssh fat01

for i in {0..19};do ( nohup bash /data/sherry/jimmy/submit.sh qualimap.sh 10 $i 1> $output/log_paired_qualimap.$i.txt 2>&1 & ) ;done

$pwd

/home/sherry/all-clean-data/qc/qualimap_pe_hisat2

conda activate rna

nohup multiqc */* -o ./ -n pe_qualimap_multiqc 1>multiqc_log 2>&1 &

# cd /home/sherry/add/cleanData/qualimap_pe_hisat2 下载pe_qualimap_multiqc.html

##Single-ended

bin=/home/sherry/all-clean-data/software/qualimap_v2.2.1/qualimap

gtf=/home/sherry/index/Bos_taurus.ARS-UCD1.2.102.gtf

#pe_bam=/data/sherry/add/1cleanData/paired_hisat2_output/

output=/home/sherry/add/cleanData/qualimap_se_hisat2

ls *.bam|while read id;do

echo $bin rnaseq --java-mem-size=40G -bam $id -gtf $gtf -outdir $output/$(basename ${id} ".hisat_se.bam")_qc -outformat PDF:HTML -oc $(basename ${id} ".hisat_se.bam")

done > qualimap_se.sh

ssh fat01

for i in {0..9};do ( nohup bash ../submit.sh qualimap_se.sh 10 $i 1> $output/log_se_qualimap.$i.txt 2>&1 & ) ;done

#Merge results

nohup multiqc */* -o ./ -n se_qualimap_multiqc 1>multiqc_log 2>&1 &

#Statistics of bam file information

#Double-ended

ls *.bam |perl -e 'while(<>){chomp;$_=~s/.hisat.bam//g;print"samtools flagstat -@ 6 $_.hisat.bam >$_.flagstat\n";}' - |less -S >flagstat.sh

nohup bash flagstat.sh 1>flag_log 2>&1 &

##Single-ended

ls *.bam |perl -e 'while(<>){chomp;$_=~s/.hisat_se.bam//g;print"samtools flagstat -@ 4 $_.hisat_se.bam >$_.flagstat\n";}' - |less -S >flagstat_se.sh

nohup bash flagstat_se.sh 1>flag_log 2>&1 &

featureCounts

vi count.sh

gtf=/home/sherry/index/Bos_taurus.ARS-UCD1.2.102.gtf

out=/data/sherry/add/cleanData/1count/

featureCounts -T 20 -t gene -g gene_id -a $gtf -o ${out}pe_all_id.txt *.bam

##

nohup bash count.sh 1>/data/sherry/add/cleanData/1count/pe_count_log 2>&1 &

cd /data/sherry/add/cleanData/1count

nohup multiqc pe_all_id.txt.summary -o ./ -n pe_all_id_qc 1>log_pe 2>&1 &

cat pe_all_id.txt | cut -f1,7- > counts.txt
